# Cancer Detection in Breast MRI Screening via Explainable AI Anomaly Detection

**DOI:** 10.1148/radiol.241629

**Published:** 2025-07-15

**Authors:** Felipe Oviedo, Anum S. Kazerouni, Philipp Liznerski, Yixi Xu, Michael Hirano, Robert A. Vandermeulen, Marius Kloft, Elyse Blum, Adam M. Alessio, Christopher I. Li, William B. Weeks, Rahul Dodhia, Juan M. Lavista Ferres, Habib Rahbar, Savannah C. Partridge

**Affiliations:** ^1^AI for Good Lab, Microsoft, 1 Microsoft Way, 4330 150th Ave NE, Redmond, WA 98052; ^2^Department of Radiology, University of Washington School of Medicine, Seattle, Wash; ^3^University of Kaiserslautern-Landau, Kaiserslautern, Germany; ^4^Berlin Institute for the Foundations of Learning and Data, Berlin, Germany; ^5^Machine Learning Group, Technical University of Berlin, Berlin, Germany; ^6^Department of Biomedical Engineering, Michigan State University, East Lansing, Mich; ^7^Fred Hutchinson Cancer Center, Seattle, Wash

## Abstract

**Background:**

Artificial intelligence (AI) models hold potential to increase the accuracy and efficiency of breast MRI screening; however, existing models have not been rigorously evaluated in populations with low cancer prevalence and lack interpretability, both of which are essential for clinical adoption.

**Purpose:**

To develop an explainable AI model for cancer detection at breast MRI that is effective in both high- and low-cancer-prevalence settings.

**Materials and Methods:**

This retrospective study included 9738 breast MRI examinations from a single institution (2005–2022), with external testing in a publicly available multicenter dataset (221 examinations). In total, 9567 consecutive examinations were used to develop an explainable fully convolutional data description (FCDD) anomaly detection model to detect malignancies on contrast-enhanced MRI scans. Performance was evaluated in three cohorts: grouped cross-validation (for both balanced [20.0% malignant] and imbalanced [1.85% malignant] detection tasks), an internal independent test set (171 examinations), and an external dataset. Explainability was assessed through pixelwise comparisons with reference-standard malignancy annotations. Statistical significance was assessed using the Wilcoxon signed rank test.

**Results:**

FCDD outperformed the benchmark binary cross-entropy (BCE) model in cross-validation for both balanced (mean area under the receiver operating characteristic curve [AUC] = 0.84 ± 0.01 [SD] vs 0.81 ± 0.01; *P* < .001) and imbalanced (mean AUC = 0.72 ± 0.03 vs 0.69 ± 0.03; *P* < .001) detection tasks. At a fixed 97% sensitivity in the imbalanced setting, mean specificity across folds was 13% for FCDD and 9% for BCE (*P* = .02). In the internal test set, FCDD outperformed BCE for balanced (mean AUC = 0.81 ± 0.02 vs 0.72 ± 0.02; *P* < .001) and imbalanced (mean AUC = 0.78 ± 0.05 vs 0.76 ± 0.01; *P* < .02) detection tasks. For model explainability, FCDD demonstrated better spatial agreement with reference-standard annotations than BCE (internal test set: mean pixelwise AUC = 0.92 ± 0.10 vs 0.81 ± 0.13; *P* < .001). External testing confirmed that FCDD performed well, and better than BCE, in the balanced detection task (AUC = 0.86 ± 0.01 vs 0.79 ± 0.01; *P* < .001).

**Conclusion:**

The developed explainable AI model for cancer detection at breast MRI accurately depicted tumor location and outperformed commonly used models in both high- and low-cancer-prevalence scenarios.

© The Author(s) 2025. Published by the Radiological Society of North America under a CC BY 4.0 license.

*Supplemental material is available for this article.*

See also the editorial by Bae and Ham in this issue.

## Graphical abstract

**Figure:**
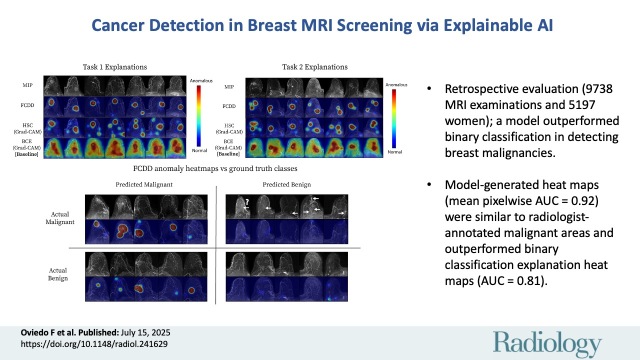

**Summary**

An anomaly detection model for breast cancer on MRI scans achieved high performance, surpassing binary classification in high- and low-cancer-prevalence scenarios and producing explanation heat maps not significantly different from radiologist annotations.

**Key Results**

■ In a retrospective evaluation (9738 contrast-enhanced breast MRI examinations; 5197 women), a deep explainable anomaly detection model outperformed binary classification in detecting breast malignancies.
■ Model-generated explanation heat maps (mean pixelwise area under the receiver operating characteristic curve [AUC] = 0.92 ± 0.10 [95% CI: 0.90, 0.93]) were not significantly different from radiologist-annotated malignant areas and substantially outperformed binary classification explanation heat maps (AUC = 0.81 ± 0.13; *P* < .001).
■ The anomaly detection model surpassed the binary classification model in cancer detection performance in grouped cross-validation and internal and external test sets, in both balanced and imbalanced detection tasks.

## Introduction

MRI has demonstrated high sensitivity for detecting breast cancer, particularly in women with elevated breast cancer risk (1). However, MRI screening has been found to produce a considerable number of false positives (1,2). Recent advances in the field have led to increased interest in the application of artificial intelligence (AI) in breast MRI, with a focus on reducing radiologist workload and false positives (3). Nevertheless, the clinical applications of AI in breast MRI are still limited, with only one U.S. Food and Drug Administration–approved algorithm for lesion diagnosis (4).

While deep learning models have been proposed for cancer detection (3) and triage (5) using MRI, they have several limitations. In the screening of patients with high cancer risk, cancers will be detected in only a small fraction (<5%) of patients (1,3). Moreover, malignant lesions exhibit significant heterogeneity (eg, in lesion type, size, and histologic characteristics) and are often underrepresented in datasets. These factors limit performance when common binary classification algorithms are used (6). Most prior breast MRI AI models were evaluated using balanced datasets of cancer and noncancer examinations (3), leading to overestimation of predictive power and potentially limiting clinical utility. Additionally, while classification models may achieve good detection performance at the examination level, they often show limited ability to highlight malignant regions within the image (7,8). This lack of interpretability limits model utility, as physicians are unable to confirm the regions used for prediction. Although various secondary AI explainability methods (9) including gradient-based mapping (10), Shapley additive explanations (5), and patch-level classification (11) have been used to generate saliency maps (heat maps of malignant areas on MRI scans) without requiring spatial annotations, these maps lack accuracy and robustness to noise in medical imaging (9,12). Model misspecification, limited saliency map resolution, and class imbalance have been shown to contribute to these issues (12,13).

The aim of this study was to address both class imbalance and explainability challenges by developing an explainable AI anomaly detection model for cancer detection on screening breast MRI scans using large, imbalanced breast MRI datasets. Anomaly detection models have demonstrated superior performance compared to conventional binary classification models because anomaly detection models focus on the majority class and effectively identify abnormal samples, even when they are underrepresented in the training data (14,15). We hypothesized that an approach using breast dynamic contrast-enhanced MRI scans as input and an anomaly detection model based on one-class classification (16) would outperform conventional models for cancer detection and provide accurate explanation heat maps without the need for lesion annotations or secondary interpretability techniques.

## Materials and Methods

This retrospective study, compliant with the Health Insurance Portability and Accountability Act and approved by the institutional review board of the Fred Hutchinson Cancer Center (approval no. 7339), used three independent patient datasets (internal and external). The primary model development dataset incorporated all consecutive breast MRI examinations (>10 000 before exclusions) performed at the University of Washington School of Medicine from July 2005 to November 2015. The requirement for informed consent was waived due to the large retrospective nature of the study. Dataset and curation details were published in a previous study (17). A separate internal enriched test dataset comprised 171 women who underwent MRI for preoperative evaluation or screening (31 cancers diagnosed at post-MRI biopsy) at the same institution between 2018 and 2022; these women were included in a prospective study (ClinicalTrials.gov: NCT03607552). External validation was performed using a publicly available multicenter breast MRI dataset that included bilateral breast MRI examinations and lesion segmentations in women with invasive breast cancer *(https://www.cancerimagingarchive.net/)* (18). From this external dataset, we included pretreatment dynamic contrast-enhanced MRI examinations in 221 women that were compatible with our semiautomated preprocessing pipeline, excluding scans requiring technical adjustment, reformatting, or expert review.

For the internal datasets, imaging characteristics (including background parenchymal enhancement [BPE], ie, the degree of contrast enhancement of normal breast tissue at MRI, categorized as minimal, mild, moderate, or marked) and outcomes were determined from clinical and pathology reports and from linked data in the regional Surveillance, Epidemiology, and End Results (or SEER) tumor registry (Table 1). Outcomes were categorized at the breast level as malignant (biopsy-confirmed cancer within 12 months before [without surgery] or after MRI) or benign (no cancer diagnosis within 12 months after MRI). Age and race data were collected from the electronic health records. For the external dataset, patient demographic data were derived from the provided metadata, using the same outcome definitions (Table S7).

**Table 1:** Patient Characteristics in the Model Development Dataset and Independent Internal Test Set

Characteristic	Model Development Dataset		Internal Test Set	
	Task 1 (Balanced Detection)	Task 2 (Imbalanced Detection)	Task 1 (Balanced Detection)	Task 2 (Imbalanced Detection)
No. of patients	5026	2808	171	123
No. of MRI examinations	9567	6310	171	123
No. of unilateral breast MIPs	17 029	11 934	342	246
Age at examination (y)*	51.5 ± 11.1	50.4 ± 10.8	48.8 ± 12.4	48.3 ± 12.1
Race^†^				
White	7776 (81.3)	5168 (81.9)	147 (86.0)	106 (86.2)
Asian	630 (6.6)	369 (5.8)	14 (8.2)	9 (7.32)
Black	318 (3.3)	166 (2.6)	1 (0.58)	1 (0.81)
Mixed race	173 (1.8)	91 (1.44)	0 (0)	0 (0)
Native American	53 (0.6)	35 (0.55)	2 (1.17)	2 (1.6)
Hawaiian or Pacific Islander	11 (0.1)	2 (0.03)	0 (0)	0 (0)
Unknown	598 (6.3)	479 (7.6)	7 (4.1)	5 (4.1)
Indication^†^				
Screening	5332 (55.7)	5332 (84.5)	123 (71.9)	123 (100)
Known cancer	3234 (33.8)	0 (0.00)	48 (28.1)	0 (0)
Diagnostic	502 (5.2)	496 (7.9)	0 (0)	0 (0)
Other	499 (5.2)	482 (7.6)	0 (0)	0 (0)
Malignant outcome^‡^	3399 (20.0)	221 (1.85)	81 (23.7)	31 (12.6)
Mammographic breast density^‡^				
Fatty	350 (2.1)	122 (1.02)	0 (0)	0 (0)
Scattered density	3408 (20.0)	2177 (18.2)	42 (12.3)	24 (9.8)
Heterogeneously dense	7298 (42.9)	5125 (42.9)	218 (63.7)	146 (59.3)
Extremely dense	1977 (11.6)	1455 (12.2)	82 (24.0)	76 (30.9)
Unknown	3966 (23.3)	2944 (24.7)	0 (0)	0 (0)
Background parenchymal enhancement^‡^				
Minimal	6814 (40.0)	5269 (44.1)	100 (29.2)	80 (32.5)
Mild	4181 (24.6)	2720 (22.8)	110 (32.2)	72 (29.3)
Moderate	1940 (11.4)	1275 (10.7)	84 (24.6)	50 (20.3)
Marked	970 (5.7)	717 (6.01)	48 (14.0)	44 (17.9)
Unknown	3121 (18.3)	1953 (16.4)	0 (0)	0 (0)

Note.—Except where indicated, data are numbers of patients, with percentages in parentheses. MIP = maximum intensity projection.

* Data are means ± SDs, calculated at the patient level.

† Calculated at the examination level.

‡ Calculated at the unilateral breast MIP level.

### MRI Acquisition and Postprocessing

MRI acquisition details are summarized in Appendix S1. All bilateral breast MRI examinations met American College of Radiology standards (19) and were performed with a 1.5- or 3-T MRI scanner using dedicated breast coils. Each MRI examination included a three-dimensional axial dynamic contrast-enhanced MRI sequence, reduced to a two-dimensional maximum intensity projection (MIP) from subtraction images (first postcontrast image minus precontrast image). Each MIP was split into two single-breast MIP images via preprocessing described previously (17) and in Appendix S3.

### Model Development

Two cancer detection tasks were defined, which involved classifying unilateral MIPs as malignant or benign in two different populations. The population for task 1 (balanced detection) included all breast MIPs, regardless of prior cancer diagnosis, while the population for task 2 (imbalanced detection) involved only MIPs without a known cancer diagnosis at the time of examination, approximating breast cancer prevalence in MRI screening (1). Grouped cross-validation was used to evaluate both detection tasks in the model development dataset. Additionally, independent evaluation was performed using the internal enriched breast MRI test set (tasks 1 and 2) and the external dataset (task 1).

To evaluate model explanation heat maps, an explanation test set of 55 women with malignancies was considered (Table 2). This holdout set from the model development dataset comprised a random stratified sample of women with Breast Imaging Reporting and Data System (or BI-RADS) 4 and 5 breast cancer detected at MRI (85% [47 of 55]), supplemented with a random sample of women with known BI-RADS 6 cancer (15% [eight of 55]). Lesion characteristics—MRI lesion type (mass vs nonmass enhancement [NME]) and cancer T stage—were obtained from clinical reports. A breast radiology fellow (E.B., with 1 year of breast imaging experience), blinded to AI predictions, retrospectively reviewed the pathology and imaging reports and annotated the malignant regions directly on the breast MIPs. Pixels within annotation were labeled abnormal; all others were labeled normal. Additional explainability validation was performed using the full external dataset with tumor segmentations.

**Table 2:** Patient Characteristics in the Explanation Test Set

Characteristic	Value
No. of patients	55 (100)
Age (y)*	53.5 ± 9.8
Race	
White	42 (76)
Asian	4 (7)
Black	2 (4)
Mixed race	1 (2)
Native American	0 (0)
Hawaiian or Pacific Islander	1 (2)
Unknown	5 (9)
Mammographic breast density	
Fatty	0 (0)
Scattered density	11 (20)
Heterogeneously dense	33 (60)
Extremely dense	5 (9)
Unknown	6 (11)
Background parenchymal enhancement	
Minimal	19 (35)
Mild	18 (33)
Moderate	9 (16)
Marked	3 (5)
Unknown	6 (11)
Indication	
Screening	47 (85)
Known cancer	8 (15)
T stage	
Tis^†^	14 (25)
T1	26 (47)
T2	10 (18)
T3	5 (9)
Lesion type	
Mass	31 (56)
NME	18 (33)
Mass and NME	4 (7.3)
Other	2 (4)
Scanner	
GE HealthCare Signa 1.5T	24 (44)
Philips Achieva 3.0T	31 (56)

Note.—Except where indicated, data are numbers of patients, with percentages in parentheses. This dataset set was a holdout dataset with reference-standard spatial annotations. For evaluation, the model was trained using all other patients in the development dataset, and explanations were generated and evaluated in this test set. NME = nonmass enhancement.

* Data are means ± SDs.

† Ductal carcinoma in situ.

### Algorithm

**Figure 1:**
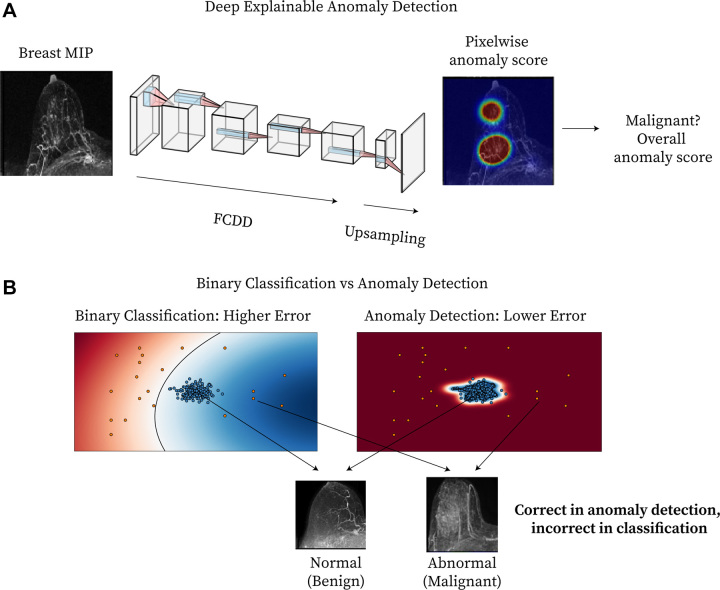
Method overview. **(A)** Diagram shows deep explainable anomaly detection at breast MRI. A maximum intensity projection (MIP) of a breast is passed to a fully convolutional neural network model trained using an explainable anomaly detection loss function (fully convolutional data description [FCDD]). The model directly outputs an upsampled heat map of the anomalous pixels (anomaly heat map). The mean anomaly score across all pixels is used to classify a case as abnormal (malignant) or not. **(B)** Diagram shows a conceptual comparison between binary classification and anomaly detection: Shading represents the learned normal (blue) and abnormal (red) feature spaces, and dots represent individual normal (blue) and abnormal (red) cases. Binary classification learns a classification boundary between normal and abnormal cases, but model performance is affected by the scarcity and variability of malignant cases. Anomaly detection focuses on robustly learning the normal class and aims to identify abnormal cases accurately under these conditions.

The model used a semisupervised anomaly detection loss function and a fully convolutional neural network to generate pixelwise and breast-level anomaly scores (Fig 1A) (16). Unlike traditional binary classification, anomaly detection models learn a robust representation of the nominal (ie, benign) feature space to better identify abnormal (ie, malignant) images (Fig 1B). The fully convolutional data description (FCDD) model (16) was used, which estimates anomaly scores and produces spatially resolved heat maps (anomaly heat maps) as model explanations, without requiring secondary interpretability techniques or spatial lesion annotations. Each heat map comprises pixelwise anomaly scores, averaged to yield a final breast-level anomaly score. Considering bilateral symmetry, we explored a variant model (FCDD-Symmetric) using the contralateral breast as the normal class center. The comparison models were binary cross-entropy (BCE) (a traditional binary classifier) and hypersphere classification (HSC) (14) (a nonexplainable anomaly detector). To compare the explainability of these models with that of FCDD, saliency maps were generated for both BCE and HSC using gradient-weighted class activation mapping, or Grad-CAM (20). Additional details are provided in Appendices S2 and S3. Code for this model is provided in the following GitHub repository: *https://github.com/microsoft/breastMRI-fcdd*.

### Statistical Analysis and Evaluation

Patient-grouped fivefold cross-validation was used to evaluate cancer detection, with each fold trained using five random seeds. Test folds for task 2 excluded MRI scans with known cancers; however, supplementing training folds with MRI scans with known cancers was observed to improve performance (Table S1). Detection performance was evaluated at three operating points: one maximizing the Youden index and two maximizing sensitivity (at 95% and 97%). Area under the receiver operating characteristic curve (AUC), specificity, sensitivity, positive predictive value (PPV), and area under the precision-recall curve (AUPR) were calculated by one author (F.O.) using the scikit-learn (version 0.24.2) module for Python (Python Software Foundation). AUPR estimates the balance between PPV and sensitivity and is useful for imbalanced classification; random prediction yields an AUPR equal to the minority class proportion (eg, cancer prevalence). Detection performance was independently validated for tasks 1 and 2 in the independent internal test set without retraining.

Model explainability was evaluated using the reference-standard spatial annotations from the explanation test set. Explanation maps were compared with the radiologist’s annotations by measuring pixelwise AUC (16) for each breast image; higher AUC indicated greater spatial consistency with the radiologist annotation. Generalization for model detection and explanation without retraining was assessed using the external multicenter dataset.

The Wilcoxon signed-rank test (21) was used for comparisons. Statistical analyses were performed by one author (F.O.) using the statsmodels (version 0.13.2) module for Python, with a significance threshold of *P* < .05.

## Results

**Figure 2:**
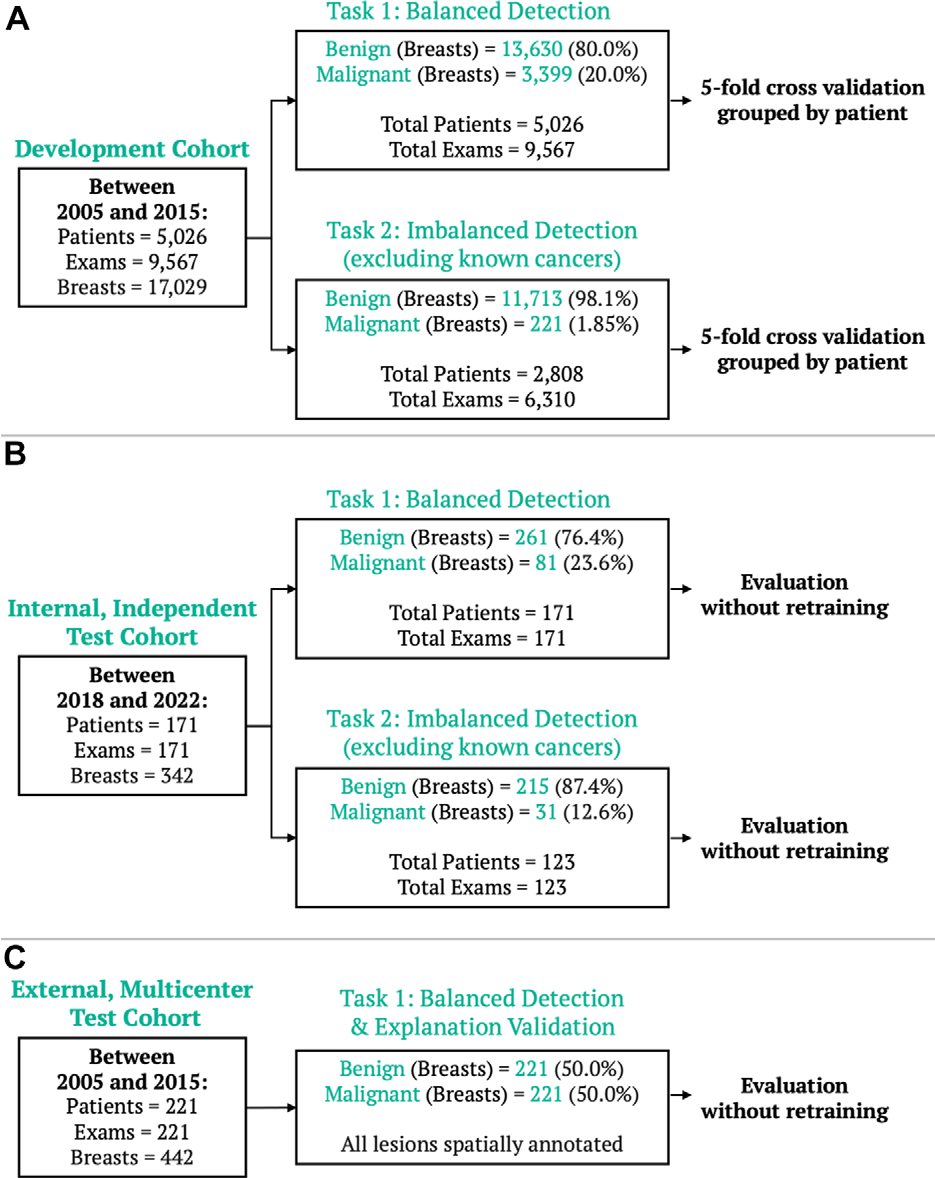
Breast MRI datasets. **(A)** The model development dataset was composed of data from 5026 patients (9567 examinations that occurred from 2005 to 2015). Two detection tasks were defined: balanced detection (which included all data) and imbalanced detection (which excluded MRI scans with known cancers). For each task, fivefold cross-validation grouped by patient was performed, preserving the original class imbalance. To evaluate the model explanations, a holdout explanation dataset, composed of malignant cases with lesions retrospectively annotated by a radiologist, was used. Evaluation was also performed on two independent datasets: **(B)** an internal dataset of 171 examinations (from 2018 to 2022), evaluated for both the balanced and imbalanced detection tasks, and **(C)** an external multicenter dataset of 221 examinations (from 2005 to 2015), evaluated for the balanced detection task and for spatial model explanations.

Of 10 185 consecutive breast MRI examinations performed in 5248 patients during the data collection period for the model development dataset, subtraction MIPs and outcomes were successfully obtained for 9567 examinations in 5026 patients (mean age, 51.5 years ± 11.1 [SD]). Examinations were excluded due to clinical (eg, postmastectomy examination or indeterminate outcome) or technical (eg, artifacts or missing or corrupted files) issues (Fig 2). Patients were predominantly White (4012 of 5026 [79.8%]) with heterogeneously or extremely dense breasts (7298 [42.9%] and 1977 [11.6%] of 17 029 unilateral breast MIPs, respectively) and minimal or mild BPE (6814 [40.0%] and 4181 [24.6%] of 17 029 unilateral breast MIPs, respectively). MRI examinations were primarily performed for screening (5332 of 9567 [55.7%]), followed by evaluation of known cancer (3234 of 9567 [33.8%]). The final model development dataset included 17 029 unilateral breast MIPs, with malignancy present in 20.0% (3399 of 17 029) for task 1 (balanced detection) and 1.85% (221 of 11 934) for task 2 (imbalanced detection) (Fig 1, Table 1).

The independent internal test set included 342 unilateral breast MIPs from 171 women (mean age, 48.8 years ± 12.4) (Table 2). Malignant outcomes were found in 23.7% (81 of 342) of unilateral breast MIPs for task 1 and 12.6% (31 of 246) for task 2 (Table 2). The external multicenter dataset included 385 publicly available pretreatment breast MRI examinations, of which 221 (442 unilateral breast MIPs) (Table S7) were compatible with our processing pipeline, representing diverse scanner platforms, demographic characteristics, and lesion types. Exclusions were due to technical issues such as Digital Imaging and Communications in Medicine, or DICOM, header inconsistencies, segmentation orientation errors, and motion artifacts.

### Cross-Validation Results

**Figure 3:**
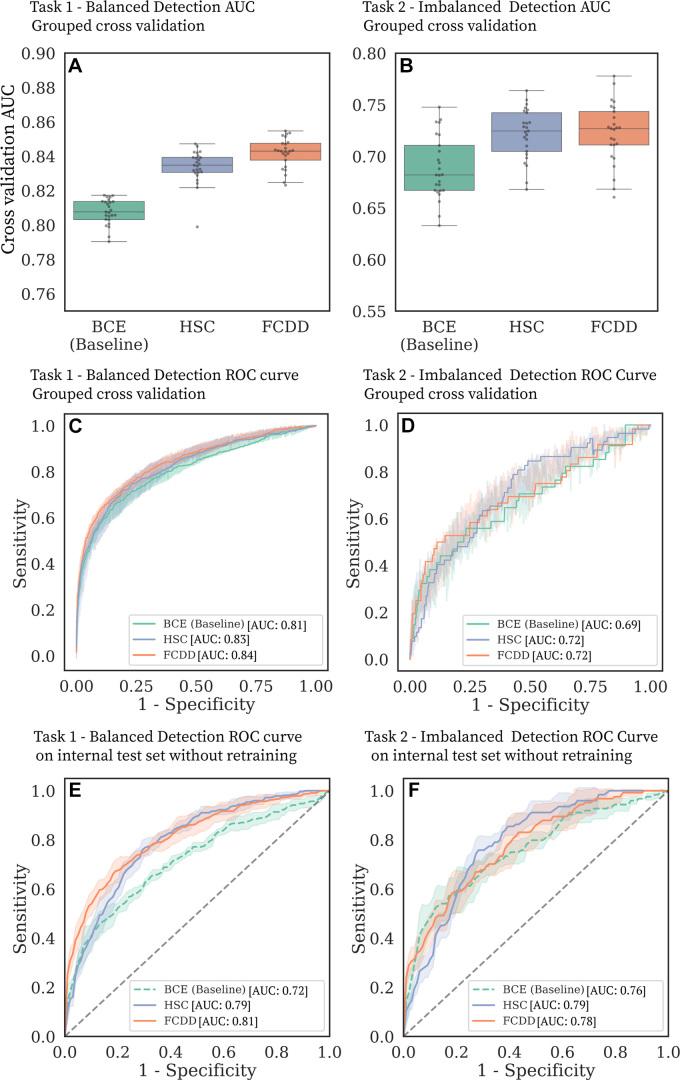
Cancer detection performance on balanced and imbalanced tasks. **(A–D)** Binary cross-entropy (BCE) is compared with the anomaly detection models hypersphere classification (HSC) and fully convolutional data description (FCDD) in the large model development dataset. **(A, B)** Box and whisker plots (box, IQR; line, median; upper whisker, point in range quartile 1 − 1.5 × IQR; lower whisker, point in range quartile 3 + 1.5 × IQR; dots, outliers) show area under the receiver operating characteristic (ROC) curve (AUC) distributions for **(A)** task 1 and **(B)** task 2 for five grouped cross-validation test folds, with five random initializations per fold. **(C, D)** ROC curves for **(C)** task 1 and **(D)** task 2 in grouped cross-validation. The shaded areas correspond to ROC curves for different test folds and random model initializations. **(E, F)** ROC curves for **(E)** task 1 and **(F)** task 2 for the independent internal test set.

Cancer detection performance for tasks 1 (balanced detection) and 2 (imbalanced detection) is summarized in Figure 3 and Table 3. In task 1, FCDD outperformed BCE, with a mean AUC of 0.84 ± 0.01 across folds and seeds (vs 0.81 ± 0.01 for BCE; *P* < .001) and a mean AUPR of 0.69 ± 0.01 (vs 0.63 ± 0.01 for BCE; *P* < .001). In task 2, HSC and FCDD were top performers; FCDD achieved a mean AUC of 0.72 ± 0.03 (vs 0.69 ± 0.03 for BCE; *P* < .001) and a mean AUPR of 0.11 ± 0.03 (vs 0.09 ± 0.03 for BCE; *P* = .007). Bootstrapped CIs and fold-wise *P* values are reported in Tables S2 and S3, respectively. FCDD-Symmetric outperformed BCE but not FCDD (Table S4, Appendix S5). Stratification by BPE (Table S5) showed reduced performance with higher BPE, especially marked BPE, though FCDD consistently outperformed other models across all BPE levels. At the Youden index (Table 3), there was no evidence of a difference in PPV between BCE and FCDD for task 1, but for task 2, the PPV of FCDD was twice that of BCE (14% ± 4 vs 7% ± 3; *P* = .005). On average, FCDD yielded 175 false positives per fold compared to 233 for BCE. At 95% and 97% sensitivity, FCDD and HSC showed significantly higher specificity than BCE in both tasks (*P* < .001 for all).

**Table 3:** Model Performance for Cancer Detection in the Model Development Dataset (Grouped Cross-Validation) and Internal Test Set

Dataset, Task, and Model	AUC*	AUPR^†^	Maximizing Youden Index			Maximizing Sensitivity	
			PPV (%)	Specificity (%)	Sensitivity (%)	Specificity at 95% Sensitivity	Specificity at 97% Sensitivity
Model development dataset							
Task 1 (balanced detection)							
BCE	0.81 ± 0.01	0.63 ± 0.01	53 ± 4	86 ± 4	63 ± 5	22 ± 3	14 ± 3
HSC	0.83 ± 0.01^‡^	0.65 ± 0.02^‡^	50 ± 3	82 ± 3	70 ± 3^‡^	30 ± 4^‡^	21 ± 3^‡^
FCDD	0.84 ± 0.01^‡^	0.69 ± 0.01^‡^	54 ± 4	85 ± 3	69 ± 4^‡^	30 ± 3^‡^	22 ± 4^‡^
Task 2 (imbalanced detection)							
BCE	0.69 ± 0.03	0.09 ± 0.03	7 ± 2	90 ± 4	39 ± 8	11 ± 7	9 ± 5
HSC	0.72 ± 0.02^‡^	0.1 ± 0.03	10 ± 5	85 ± 11	44 ± 14	17 ± 8^‡^	13 ± 6^‡^
FCDD	0.72 ± 0.03^‡^	0.11 ± 0.03^‡^	14 ± 4^‡^	93 ± 7	32 ± 13	17 ± 9^‡^	13 ± 8^‡^
Independent internal test set							
Task 1 (balanced detection)							
BCE	0.72 ± 0.02	0.52 ± 0.04	38 ± 1	66 ± 5	67 ± 5	12 ± 9	6 ± 6
HSC	0.79 ± 0.02^‡^	0.55 ± 0.05	45 ± 2^‡^	72 ± 1^‡^	76 ± 4^‡^	35 ± 6^‡^	26 ± 10^‡^
FCDD	0.81 ± 0.02^‡^	0.66 ± 0.03^‡^	51 ± 8^‡^	77 ± 9^‡^	74 ± 7^‡^	33 ± 13^‡^	24 ± 12^‡^
Task 2 (imbalanced detection)							
BCE	0.76 ± 0.01	0.40 ± 0.05	26 ± 5	69 ± 9	73 ± 6	24 ± 13	5 ± 3
HSC	0.79 ± 0.03^‡^	0.36 ± 0.06	33 ± 6^‡^	81 ± 11^‡^	60 ± 3	42 ± 11^‡^	29 ± 7^‡^
FCDD	0.78 ± 0.05^‡^	0.47 ± 0.06^‡^	30 ± 8	73 ± 14	71 ± 17	34 ± 14^‡^	25 ± 14^‡^

Note.—Data are presented as means ± SDs. The model operating point was chosen by maximizing the Youden index or sensitivity (at 95% or 97%). AUC = area under the receiver operating characteristic curve, AUPR = area under the precision-recall curve, BCE = binary cross-entropy, FCDD = fully convolutional data description, HSC = hypersphere classification, PPV = positive predictive value.

* Baseline AUC for a random guess is 0.5 for both tasks 1 and 2 in both the model development dataset and the internal test set.

† Baseline AUPR for random guess is 0.2 in the model development dataset and 0.24 in the internal test set for task 1 and 0.0185 in the model development dataset and 0.126 in the internal test set for task 2, corresponding to the prevalence of malignancies in each dataset (Table 2).

‡ Instances where an anomaly detection method (FCDD or HSC) outperformed BCE with *P* < .05.

**Figure 4:**
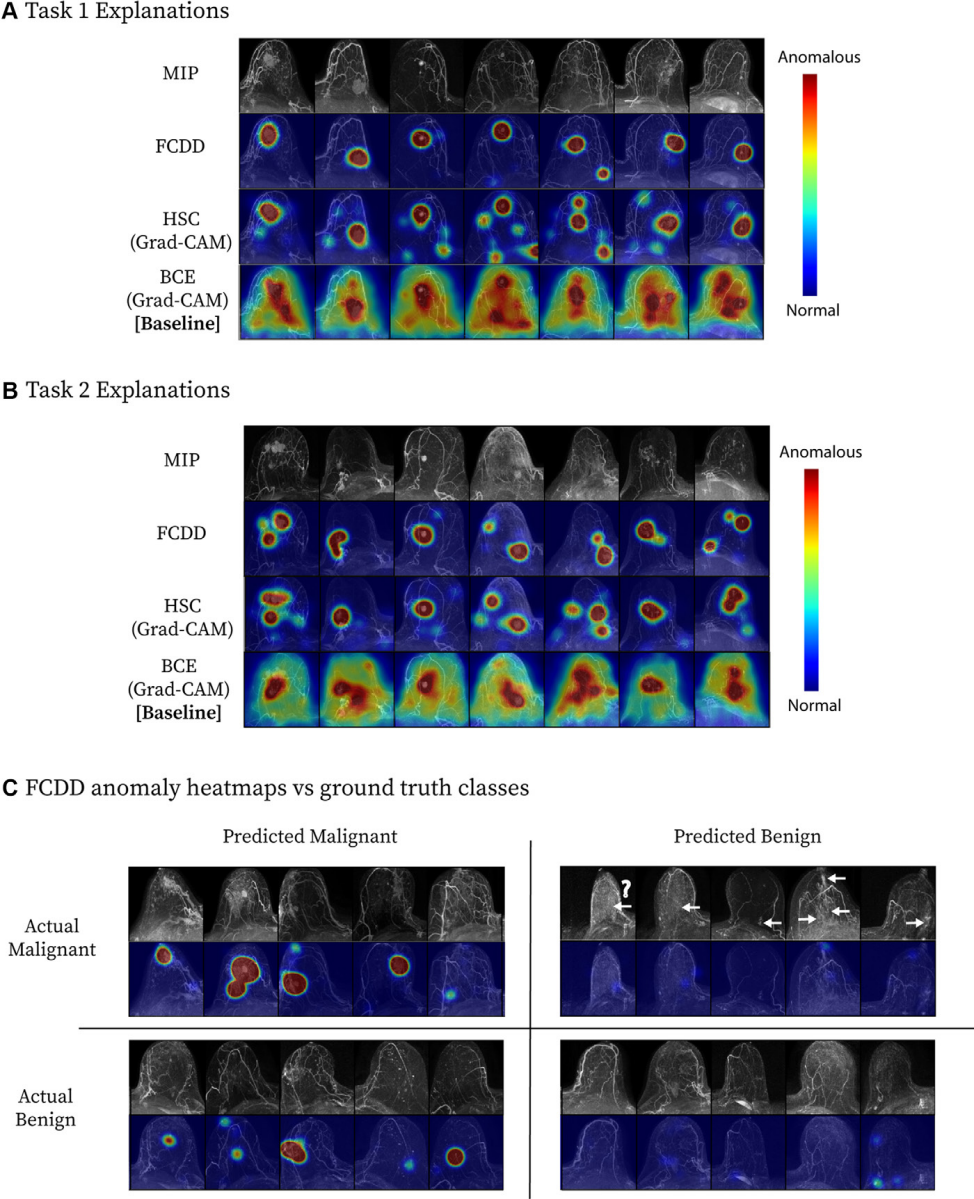
Model explanation heat maps for a cross-validation test fold. **(A, B)** Explanation maps for a random sample of maximum intensity projections (MIPs) in **(A)** task 1 and **(B)** task 2. Fully convolutional data description (FCDD) produces a more specific heat map than those computed for binary cross-entropy (BCE) and hypersphere classification (HSC). **(C)** Confusion matrix of representative FCDD anomaly heat maps for randomly sampled cases, comparing predictions and actual classes. For breasts with malignancies, locations of missed cancers (ie, on MIPs that were predicted to be benign by FCDD) are indicated by arrows. (The question mark denotes a known ductal carcinoma in situ that did not exhibit enhancement at MRI, which may have been due to a high level of background parenchymal enhancement; a common factor of missed cancers was low visibility on the MIP images.) Following the best practices delineated in Liznerski et al (16) and described in Appendix S3, the color scales of the explanation maps in **A** and **B** are locally normalized (per breast) to facilitate comparisons between models, whereas the explanation maps in **C** are globally normalized (across all the cases in the test set) to facilitate comparisons across the cases for the same model. Grad-CAM = gradient-weighted class activation mapping.

Explanation maps (Fig 4) showed that FCDD anomaly heat maps were more spatially specific than BCE saliency maps (Fig 4A, 4B). HSC maps resembled FCDD maps and were more precise than BCE maps (Appendix S4). False positives often overestimated anomalous regions, while false negatives included cancers obscured by BPE or not readily visible on MIPs (Fig 4C).

### Independent Test Set Results

Model evaluation on the internal test set without retraining showed performance comparable to cross-validation in the development dataset (Fig 3E, 3F; Table 3). FCDD and HSC provided comparable cancer detection performance, both outperforming BCE in tasks 1 and 2. FCDD achieved a mean AUC of 0.81 ± 0.02 (95% CI: 0.81, 0.82) for task 1 (vs 0.72 ± 0.02 for BCE; *P* < .001) and 0.78 ± 0.05 (95% CI: 0.77, 0.79) for task 2 (vs 0.76 ± 0.01 for BCE; *P* = .02).

### Explanation Heat Map Evaluation

**Figure 5:**
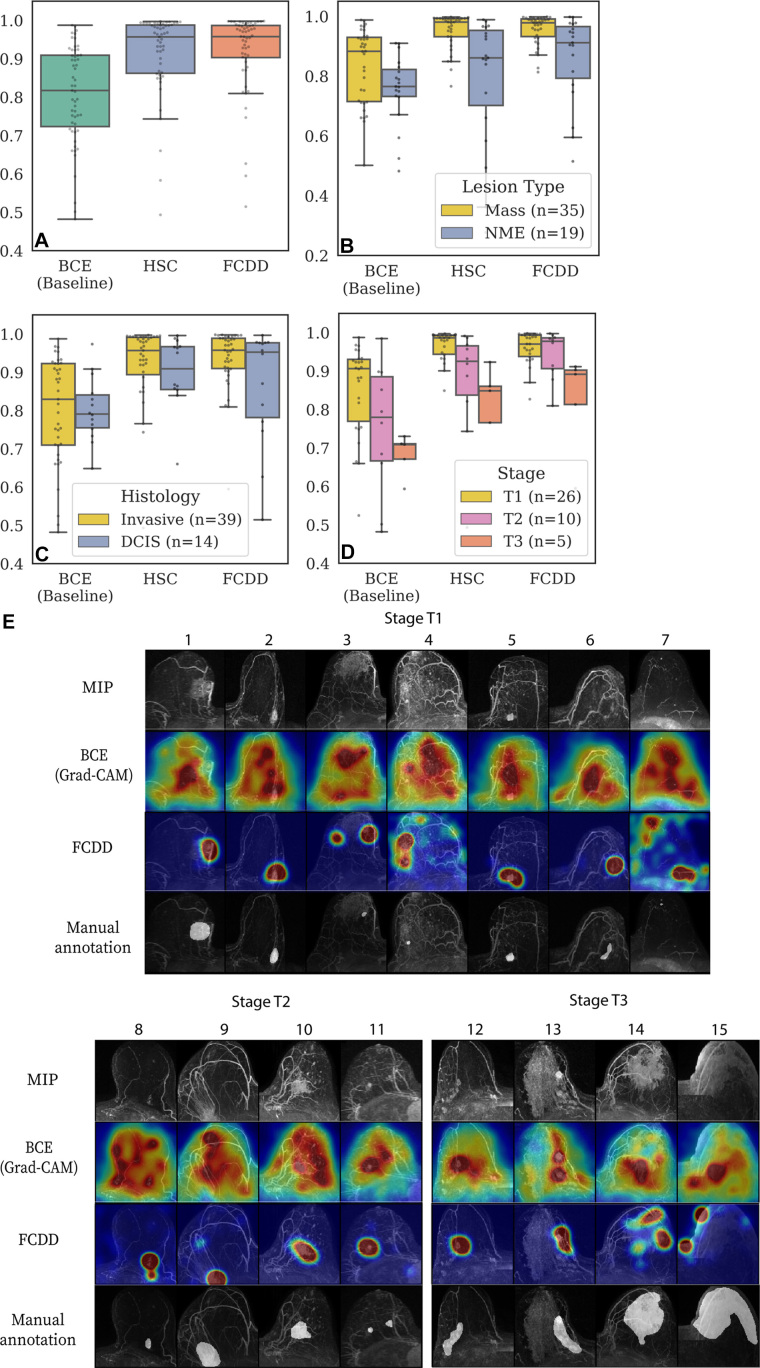
Validation of model explanation heat maps in the lesion-segmented explanation test set. **(A–D)** Box and whisker plots (box, IQR; line, median; upper whisker, point in range quartile 1 − 1.5 × IQR; lower whisker, point in range quartile 3 + 1.5 × IQR; dots, outliers) show pixelwise area under the receiver operating characteristic curve (AUC) for three models—binary cross-entropy (BCE), hypersphere classification (HSC), and fully convolutional data description (FCDD)—using the radiologist retrospective annotation as the reference standard. Each dot corresponds to the pixelwise AUC between an explanation heat map and the radiologist annotation on a breast image. Data are shown for **(A)** all images in the explanation test set, **(B)** images stratified by lesion type (mass or nonmass enhancement [NME]), **(C)** images stratified by invasive versus ductal carcinoma in situ (DCIS), and **(D)** images stratified by T stage for invasive cases. Cases with multiple lesions of different types and histologic characteristics in the same breast were excluded from **B** (one case) and **C** (two cases). **(E)** Maximum intensity projections (MIPs), BCE saliency maps, FCDD anomaly maps, and radiologist annotations are shown for a random sample of test cases with stage T1, T2, and T3 lesions. All explanation maps are locally normalized. Breasts with small lesions (eg, case 7) or multiple lesions (eg, cases 11 and 12) tended to have worse explanations. Larger stage T3 lesions (eg, cases 14 and 15) tended to be poorly explained by the BCE maps; although FCDD maps were more consistent, they tended to underestimate the size of the affected region.

Pixelwise AUCs from the internal explanation test set are shown in Figure 5A (patient characteristics provided in Table 2). FCDD (mean AUC = 0.92 ± 0.10 [95% CI: 0.90, 0.93]) outperformed BCE (mean AUC = 0.81 ± 0.13; *P* < .001). HSC performed similarly to FCDD (mean AUC = 0.90 ± 0.15; *P* < .001 vs BCE), but with higher variability. FCDD-Symmetric showed slightly higher AUC than FCDD, but with greater variance due to the per-patient reference (Appendix S5, Fig S4).

Pixelwise AUCs were higher for masses than for NME (Fig 5B) and for invasive versus in situ cancers (Fig 5C). FCDD had higher median AUCs than BCE for all lesion and histologic types but showed wider IQRs than BCE for NME and in situ cancers. Among invasive cancers, both FCDD and HSC outperformed BCE across T stages (Fig 5D), though AUCs declined with increasing stage for all models.

FCDD anomaly maps and BCE saliency maps were compared with reference-standard radiologist annotations for a random subset of stage T1, T2, and T3 malignant examinations (Fig 5E). FCDD outputs closely matched reference annotations, especially for stage T1 and T2 cases, with better spatial precision and accuracy than BCE. HSC comparisons are included in Appendix S4 and Figure S3.

### External Evaluation in a Multicenter Dataset

Without model retraining, the performance of FCDD in the balanced detection task (mean AUC = 0.86 ± 0.01 [95% CI: 0.85, 0.86]; mean AUPR = 0.88 ± 0.02 [95% CI:0.86, 0.89]) was substantially better (*P* < .001) than that of HSC (mean AUC = 0.81 ± 0.01; mean AUPR = 0.81 ± 0.01) and BCE (mean AUC = 0.79 ± 0.01; mean AUPR = 0.83 ± 0.01) (Appendix S6, Table S8). At the Youden index, FCDD also outperformed BCE in PPV, sensitivity, and specificity (Table S8). At 97% sensitivity, FCDD had higher specificity than BCE (12% ± 3 [26 of 221] vs 10% ± 5 [22 of 221]; *P* = .009) (Table S8). Explanation maps had significantly greater agreement with the reference annotations for FCDD (pixelwise mean AUC = 0.85 ± 0.13 [95% CI: 0.83, 0.87]) than for BCE (pixelwise mean AUC = 0.55 ± 0.12) and HSC (pixelwise mean AUC = 0.76 ± 0.15) (*P* < .001 for both; Fig S5).

### Past Studies Comparison

Table 4, adapted from the review by Adam et al (3), summarizes studies of deep learning models in breast MRI with more than 300 patients. For balanced detection, FCDD performance aligns well with that of prior studies (AUCs, 0.82–0.92) (3) while using larger and more diverse evaluation sets, and offers the added benefit of explainability without requiring reference-standard annotations. For imbalanced detection, benchmarks were drawn from radiologist performance reported in a large retrospective study by Lee et al (22) with real-world cancer prevalence at screening MRI (2.2% for 8387 total examinations). FCDD showed comparable PPV and specificity (Table 3) to radiologist performance (PPV, 19% specificity, 83%), albeit with lower sensitivity.

**Table 4:** Representative Prior Models

Study	Balanced or Imbalanced	No. of Patients	No. of Malignant Breasts or Lesions	Bounding Box or Contour	Heat Map	Cross-Validation	Model AUC
Antropova et al, 2018 (26)	Balanced	690	478	Bounding box	No	5-fold	0.84*
Ayatollahi et al, 2021 (27)	Balanced	462	365	Bounding box	No	10-fold	0.90
Haarburger et al, 2019 (28)	Balanced	408	305	Coarse localization	Yes	5-fold	0.82*
Herent et al, 2019 (29)	Balanced	335	123	Bounding box	Yes	NA	0.82
Hu et al, 2020 (30)	Balanced	616	728	NA	NA	5-fold	0.87
Jing et al, 2022 (23)	Imbalanced	488	118 (training set), 55 (test set)	None	Yes	No, holdout	0.81
Liu et al, 2022 (2)	Balanced	438	NA	Bounding box	No	5-fold	0.92
Truhn et al, 2019 (31)	Balanced	447	787	Contour	No	10-fold	0.88
Verburg et al, 2022 (5)	Imbalanced	4581	77	None	Yes	Per hospital	0.83*^†^
Zhou et al, 2019 (32)	Balanced	307	206	Bounding box	Yes	NA	0.86
Current study							
Task 1	Balanced	5026	3399^‡^	None	Yes	5-fold	0.84
Task 2	Imbalanced	2808	221^‡^	None	Yes	5-fold	0.72
External dataset	Balanced	221	221^‡^	None	Yes	External holdout	0.86

Note.—Known studies with more than 300 patients are presented. In the current study, the FCDD model was evaluated on a substantially larger dataset than the previous studies, as well as on an external test dataset. AUC = area under the receiver operating characteristic curve, NA = not applicable. (Table adapted, under a CC BY 4.0 license, from reference 3.)

* Mean AUC.

† Lesion versus no lesion, 838 lesions in dataset.

‡ Malignant breasts.

## Discussion

Our study revealed that anomaly detection was superior to traditional binary classification in balanced and imbalanced cancer detection tasks. In balanced detection (task 1), the fully convolutional data description (FCDD) model achieved a competitive mean area under the receiver operating characteristic curve (AUC) of 0.84 despite the fact that its performance was evaluated in a dataset that was 10 times larger than that of most prior studies (3). Furthermore, FCDD demonstrated good performance in imbalanced detection (task 2), achieving a mean AUC of 0.72 and an area under the precision-recall curve (AUPR) of 0.11 (five times the random AUPR baseline). To our knowledge, this is the first evaluation of a model in a large, low-prevalence breast cancer MRI dataset. For both tasks, performance was generally reduced at higher background parenchymal enhancement levels, and consideration of bilateral symmetry did not further improve FCDD model performance (FCDD-Symmetric model). At the Youden index, FCDD achieved twice the positive predictive value at similar sensitivity and specificity as the binary classification model (binary cross-entropy [BCE]) while reducing the number of false-positive predictions by an average of 25% (mean decrease of 58 of 233 false positives compared with BCE). At 95% and 97% sensitivity operating points, the anomaly detection models (hypersphere classification and FCDD) were superior to BCE and showed the potential to increase the number of examinations correctly discarded (ie, de-escalating or reducing urgency for radiologist review) by 50% or more (providing more than 50% higher specificity compared with BCE of both operating points).

Regarding potential integration into radiology workflows, our model could be used to quickly exclude normal scans for triage purposes and to improve reading efficiency. Furthermore, anomaly heat maps can highlight areas of potential concern, in addition to providing a “sanity check” for model predictions, allowing radiologists to focus more quickly on regions most likely to contain malignant pathology. The model by Jing et al (23), which had similar aims and comparable performance (AUC, 0.81; specificity, 18%; sensitivity, 98%) to the FCDD model in a balanced detection setting, was estimated to reduce radiologist workload by 15.7%. We would expect similar improvements, along with superior performance in the imbalanced setting, for the FCDD model. However, as shown for prior computer-aided detection tools (24), careful integration of explainable AI models into the radiologist workflow is crucial to avoid bias or overreliance on saliency maps.

Compared with the explanation maps of the other models, the FCDD maps demonstrated higher specificity and spatial accuracy, both desirable features for model outputs to be useful to radiologists (13). Pixelwise AUCs were generally lower for NME, in situ cancers, and stage T3 invasive cancers. NME is often challenging to differentiate from physiologic BPE, and ductal carcinoma in situ often presents as NME (25). This reduced conspicuity, in addition to the upsampling procedure of FCDD (Fig 1) (16), likely contributes to poorer explainable AI performance for NME and in situ cancers. We hypothesize that lower pixelwise AUCs in larger stage T3 cancers may have resulted from the limited number and diversity of large lesions in the training set.

Model evaluation in the independent internal set further confirmed the superiority of FCDD in cancer detection across both tasks, with AUC, AUPR, and other metrics mirroring those observed in the larger and more realistic (ie, not cancer-enriched) model development dataset. Stratification by BPE confirmed that FCDD surpassed BCE regardless of BPE level (Table S6). Similarly, external testing using a publicly available multicenter trial dataset (18) confirmed the generalizability of the FCDD model for both detection and explanation. The AUC for the FCDD model in the external dataset (Table S8) was comparable to the best model performance in the balanced setting (Table 3). Importantly, this external dataset included predominantly patients with larger stage 2 or 3 invasive cancers, which are easier for the FCDD model to detect.

We recognize various limitations of our study. The detection performance was reduced by the model’s using only two-dimensional subtraction MIPs. Although the FCDD loss function can be extended to include a full MRI examination or clinical variables, explainability might be reduced, as a physician would need to assess a larger number of explanations, and the upsampling procedure in higher dimensions might generate unexpected artifacts. Furthermore, model performance might be reduced when only subtle abnormalities are visible on the MRI scans or when the benign class has high variability and is difficult to learn (6). Finally, our task 1 and task 2 datasets were limited by a lack of lesion-level annotations and information (eg, tumor histologic characteristics, grade, molecular subtype, and benign vs malignant designation) and by the fact that only the explanation test set was annotated retrospectively.

In conclusion, we successfully developed an explainable fully convolutional data description (FCDD) model for cancer detection on breast MRI scans. Our findings demonstrate that the FCCD model provides an excellent compromise between explainability and detection power: Model-generated explanation maps closely matched reference tumor locations, and the model outperformed commonly used models in both high- and low-cancer-prevalence scenarios. In future work, the FCDD model should be evaluated in larger lesion-annotated datasets and prospective studies to assess its potential for accelerating breast MRI interpretation workflows.
