# Supplementary material for: Cancer Detection in Breast MRI Screening via Explainable AI Anomaly Detection
Source: Radiology. Author manuscript; Available in PMC 2026 May 29. (PMC12314762; doi:10.1148/radiol.241629)
Supplement: Supplementary Materials [file NIHMS2179190-supplement-Supplementary_Materials.pdf]

Supplemental materials undergo peer review but are not copyedited, and may include typographical errors.

© The Author(s) 2025. Published by the Radiological Society of North America under a CC BY 4.0 license.
10.1148/radiol.241629

Table S1: *Cross-validation performance for BCE, HSC and FCDD comparing the training folds in the model development dataset with and without known cancers. The test folds remain unchanged by each task.*

Task	Model	AUC (Mean±SD across test folds)
**Task 1 Balanced Detection**	BCE **without known** cancers in training folds	0.77±0.02
	BCE **with known** cancers in training folds	0.81±0.01
	HSC **without known** cancers in training folds	0.79±0.01
	HSC **with known** cancers in training folds	0.83±0.01
	FCDD **without known** cancers in training folds	0.79±0.02
	FCDD **with known** cancers in training folds	0.84±0.01
**Task 2 Imbalanced Detection**	BCE **without known** cancers in training folds	0.64±0.02
	BCE **with known** cancers in training folds	0.69±0.03
	HSC **without known** cancers in training folds	0.68±0.02
	HSC **with known** cancers in training folds	0.72±0.02
	FCDD **without known** cancers in training folds	0.69±0.04
	FCDD **with known** cancers in training folds	0.72±0.03

Abbreviations: BCE: binary cross entropy, HSC: hypersphere classification, FCDD: fully convolutional data description, AUC: area under the receiver operating curve, AUPR: area under the precision recall curve, PPV: positive predictive value SD: standard deviation.

Table S2: *95% bootstrapped confidence intervals for each metric. Cells in bold correspond to metrics that surpass BCE with P<.05).*

				Maximizing Youden's Index			Maximizing Sensitivity	
**Task**	**Model**	**AUC**	**AUPR**	**PPV**	**Specificity**	**Sensitivity**	**Specificity @95% Sensitivity**	**Specificity @97% Sensitivity**
**Task 1 Balanced Detection**	BCE	0.808 (0.805, 0.81)	0.631 (0.627, 0.636)	0.529 (0.513, 0.546)	0.857 (0.843, 0.871)	0.627 (0.609, 0.645)	0.219 (0.209, 0.23)	0.142 (0.133, 0.15)
	HSC	**0.834 (0.83, 0.837)**	**0.654 (0.645, 0.661)**	0.498 (0.486, 0.509)	0.822 (0.81, 0.832)	**0.702 (0.69, 0.714)**	**0.303 (0.29, 0.32)**	**0.208 (0.197, 0.22)**
	FCDD	**0.842 (0.838, 0.845)**	**0.685 (0.681, 0.69)**	0.54 (0.526, 0.553)	0.852 (0.839, 0.862)	**0.688 (0.675, 0.702)**	**0.3 (0.287, 0.31)**	**0.218 (0.204, 0.23)**
**Task 2 Imbalanced Detection**	BCE	0.689 (0.677, 0.7)	0.093 (0.083, 0.099)	0.073 (0.066, 0.081)	0.9 (0.885, 0.914)	0.391 (0.36, 0.421)	0.106 (0.084, 0.13)	0.086 (0.069, 0.1)
	HSC	**0.722 (0.712, 0.731)**	0.098 (0.086, 0.11)	0.1 (0.053, 0.18)	0.854 (0.808, 0.897)	0.44 (0.356, 0.52)	**0.165 (0.139, 0.19)**	**0.134 (0.12, 0.16)**
	FCDD	**0.722 (0.711, 0.733)**	**0.111 (0.101, 0.123)**	**0.142 (0.102, 0.188)**	0.925 (0.89, 0.956)	0.321 (0.25, 0.394)	**0.166 (0.134, 0.2)**	0.131 (0.1, 0.16)

Abbreviations: BCE: binary cross entropy, HSC: hypersphere classification, FCDD: fully convolutional data description, AUC: area under the receiver operating curve, AUPR: area under the precision recall curve, PPV: positive predictive value SD: standard deviation.

Supplemental materials undergo peer review but are not copyedited, and may include typographical errors.

Table S3: *P values for individual folds comparing FCDD to BCE based on repeated model initializations. Values in bold are statistically significant (P<.05). Abbreviations: AUC: area under the receiver operating curve, AUPR: area under the precision recall curve.*

		Test Fold	P Value
**AUC**	**Task 1 - Balanced**	0	**1.26E-04**
**AUC**	**Task 1 - Balanced**	1	**2.17E-03**
**AUC**	**Task 1 - Balanced**	4	**1.62E-04**
**AUC**	**Task 1 - Balanced**	2	**5.61E-05**
**AUC**	**Task 1 - Balanced**	3	**1.24E-03**
**AUC**	**Task 2 - Imbalanced**	0	2.11E-01
**AUC**	**Task 2 - Imbalanced**	1	8.42E-01
**AUC**	**Task 2 - Imbalanced**	4	**4.17E-02**
**AUC**	**Task 2 - Imbalanced**	2	**1.54E-03**
**AUC**	**Task 2 - Imbalanced**	3	**8.24E-03**
**AUPR**	**Task 1 - Balanced**	0	**3.17E-05**
**AUPR**	**Task 1 - Balanced**	1	**5.04E-04**
**AUPR**	**Task 1 - Balanced**	4	**4.29E-04**
**AUPR**	**Task 1 - Balanced**	2	**8.25E-05**
**AUPR**	**Task 1 - Balanced**	3	**3.53E-04**
**AUPR**	**Task 2 - Imbalanced**	0	3.05E-01
**AUPR**	**Task 2 - Imbalanced**	1	**1.92E-02**
**AUPR**	**Task 2 - Imbalanced**	4	**1.77E-01**
**AUPR**	**Task 2 - Imbalanced**	2	**1.09E-02**
**AUPR**	**Task 2 - Imbalanced**	3	**4.43E-04**

Supplemental materials undergo peer review but are not copyedited, and may include typographical errors.

Table S4: *Detection metrics for FCDD-Symmetric (highlighted in gray) compared to other models. Cells in bold correspond to models that are statistically significant compared to the BCE baseline (P<.05); data is presented as mean ± SD. FCDD-Symmetric shows similar performance as FCDD with more variability. Thus, we prefer the simpler FCDD model.*

				Maximizing Youden's Index			Maximizing Sensitivity	
**Task**	**Model**	**AUC**	**AUPR**	**PPV**	**Specificity**	**Sensitivity**	**Specificity @95% Sensitivity**	**Specificity @97% Sensitivity**
**Task 1 Balanced Detection**	BCE	0.808±0.01	0.631±0.01	0.529±0.04	0.857±0.04	0.627±0.05	0.219±0.03	0.142±0.03
	HSC	**0.834±0.01**	**0.654±0.02**	0.498±0.03	0.822±0.03	**0.702±0.03**	**0.303±0.04**	**0.208±0.03**
	FCDD	**0.842±0.01**	**0.685±0.01**	0.54±0.04	0.852±0.03	**0.688±0.04**	**0.3±0.03**	**0.218±0.04**
	**FCDD-SYMMETRIC**	**0.842±0.01**	**0.684±0.01**	0.516±0.04	0.833±0.03	**0.705±0.04**	0.298±0.04	0.2±0.03
**Task 2 Imbalanced Detection**	BCE	0.689±0.03	0.093±0.03	0.073±0.02	0.9±0.04	0.391±0.08	0.106±0.07	0.086±0.05
	HSC	**0.722±0.02**	0.098±0.03	0.1±0.05	0.854±0.11	0.44±0.14	**0.165±0.08**	**0.134±0.06**
	FCDD	**0.722±0.03**	**0.111±0.03**	**0.142±0.05**	0.925±0.07	0.321±0.13	**0.166±0.09**	0.131±0.08
	**FCDD-SYMMETRIC**	**0.721±0.03**	**0.112±0.03**	0.206±0.12	0.9±0.10	0.373±0.17	**0.164±0.10**	0.135±0.09

Abbreviations: BCE: binary cross entropy, HSC: hypersphere classification, FCDD: fully convolutional data description, AUC: area under the receiver operating curve, AUPR: area under the precision recall curve, PPV: positive predictive value.

Supplemental materials undergo peer review but are not copyedited, and may include typographical errors.

***Table S5:** AUC, AUPR and case count across test folds stratified by BPE level*

Task	Model	BPE	N Malignant Breasts (Mean ± SD per Fold)	N Total Breasts (Mean ± SD per Fold)	Cancer Prevalence (Malignant Breasts/Total Breasts)	AUC	AUPR
Task 1 - Balanced	BCE	Minimal	225.8 ± 12.1	1363.4 ± 73.9	16.60%	0.817 ± 0.01	0.632 ± 0.02
		Mild	219.8 ± 13.6	836.2 ± 33.9	26.30%	0.836 ± 0.02	0.73 ± 0.04
		Moderate	98.8 ± 12.2	388.0 ± 29.8	25.50%	0.779 ± 0.03	0.634 ± 0.06
		Marked	38.6 ± 7.4	194.0 ± 24.4	19.90%	0.663 ± 0.04	0.443 ± 0.07
	HSC	Minimal	225.8 ± 12.1	1363.4 ± 73.9	16.60%	0.848 ± 0.01	0.672 ± 0.03
		Mild	219.8 ± 13.6	836.2 ± 33.9	26.30%	0.86 ± 0.02	0.749 ± 0.04
		Moderate	98.8 ± 12.2	388.0 ± 29.8	25.50%	0.813 ± 0.04	0.684 ± 0.06
		Marked	38.6 ± 7.4	194.0 ± 24.4	19.90%	0.714 ± 0.04	0.466 ± 0.1
	FCDD	Minimal	225.8 ± 12.1	1363.4 ± 73.9	16.60%	0.853 ± 0.01	0.686 ± 0.03
		Mild	219.8 ± 13.6	836.2 ± 33.9	26.30%	0.864 ± 0.01	0.77 ± 0.03
		Moderate	98.8 ± 12.2	388.0 ± 29.8	25.50%	0.819 ± 0.03	0.706 ± 0.06
		Marked	38.6 ± 7.4	194.0 ± 24.4	19.90%	0.731 ± 0.04	0.506 ± 0.07
Task 2 - Imbalanced	BCE	Minimal	14.4 ± 3.8	1053.8 ± 57.2	1.40%	0.613 ± 0.06	0.082 ± 0.04
		Mild	12.6 ± 2.7	544.0 ± 33.2	2.30%	0.738 ± 0.06	0.171 ± 0.07
		Moderate	6.6 ± 2.6	255.0 ± 23.3	2.60%	0.761 ± 0.16	0.225 ± 0.2
		Marked	3.4 ± 1.2	143.4 ± 24.2	2.40%	0.542 ± 0.12	0.072 ± 0.06
	HSC	Minimal	14.4 ± 3.8	1053.8 ± 57.2	1.40%	0.683 ± 0.09	0.081 ± 0.06
		Mild	12.6 ± 2.7	544.0 ± 33.2	2.30%	0.77 ± 0.04	0.193 ± 0.06
		Moderate	6.6 ± 2.6	255.0 ± 23.3	2.60%	0.759 ± 0.17	0.242 ± 0.17
		Marked	3.4 ± 1.2	143.4 ± 24.2	2.40%	0.569 ± 0.19	0.087 ± 0.11
	FCDD	Minimal	14.4 ± 3.8	1053.8 ± 57.2	1.40%	0.668 ± 0.09	0.081 ± 0.05
		Mild	12.6 ± 2.7	544.0 ± 33.2	2.30%	0.774 ± 0.04	0.216 ± 0.05
		Moderate	6.6 ± 2.6	255.0 ± 23.3	2.60%	0.749 ± 0.16	0.272 ± 0.23
		Marked	3.4 ± 1.2	143.4 ± 24.2	2.40%	0.61 ± 0.14	0.141 ± 0.15

Abbreviations: BCE: binary cross entropy, HSC: hypersphere classification, FCDD: fully convolutional data description, AUC: area under the receiver operating curve, AUPR: area under the precision recall curve, SD: standard deviation.

Supplemental materials undergo peer review but are not copyedited, and may include typographical errors.

Table S6: *AUC, AUPR and case count stratified by BPE in the independent, internal test set, evaluated without retraining.*

Task	Model	BPE	N Malignant Breasts	N Total Breasts	Cancer Prevalence (Malignant Breasts/Total Breasts)	AUC (mean ± SD)	AUPR (mean ± SD)
Task 1 - Balanced	BCE	Minimal	22	100	22.00%	0.767 ± 0.026	0.628 ± 0.055
		Mild	32	110	29.09%	0.66 ± 0.022	0.494 ± 0.032
		Moderate	24	84	28.57%	0.716 ± 0.04	0.569 ± 0.077
		Marked	3	48	6.25%	0.793 ± 0.12	0.2 ± 0.13
	HSC	Minimal	22	100	22.00%	0.705 ± 0.038	0.44 ± 0.081
		Mild	32	110	29.09%	0.85 ± 0.021	0.653 ± 0.039
		Moderate	24	84	28.57%	0.793 ± 0.036	0.676 ± 0.049
		Marked	3	48	6.25%	0.743 ± 0.09	0.128 ± 0.038
	FCDD	Minimal	22	100	22.00%	0.772 ± 0.036	0.635 ± 0.042
		Mild	32	110	29.09%	0.836 ± 0.017	0.722 ± 0.051
		Moderate	24	84	28.57%	0.82 ± 0.054	0.719 ± 0.058
		Marked	3	48	6.25%	0.826 ± 0.077	0.38 ± 0.17
Task 2 - Imbalanced	BCE	Minimal	12	80	15.00%	0.827 ± 0.035	0.62 ± 0.11
		Mild	12	72	16.67%	0.691 ± 0.013	0.368 ± 0.046
		Moderate	6	50	12.00%	0.73 ± 0.051	0.355 ± 0.13
		Marked	1	44	2.27%	0.674 ± 0.21	0.0823 ± 0.11
	HSC	Minimal	12	80	15.00%	0.731 ± 0.059	0.367 ± 0.087
		Mild	12	72	16.67%	0.833 ± 0.047	0.43 ± 0.07
		Moderate	6	50	12.00%	0.858 ± 0.036	0.551 ± 0.031
		Marked	1	44	2.27%	0.564 ± 0.24	0.0301 ± 0.013
	FCDD	Minimal	12	80	15.00%	0.811 ± 0.054	0.565 ± 0.082
		Mild	12	72	16.67%	0.771 ± 0.04	0.492 ± 0.099
		Moderate	6	50	12.00%	0.772 ± 0.12	0.58 ± 0.075
		Marked	1	44	2.27%	0.82 ± 0.096	0.0673 ± 0.03

Abbreviations: BCE: binary cross entropy, HSC: hypersphere classification, FCDD: fully convolutional data description, BPE: background parenchymal enhancement, AUC: area under the receiver operating curve, AUPR: area under the precision recall curve, SD: standard deviation.

Supplemental materials undergo peer review but are not copyedited, and may include typographical errors.

Table S7: *Clinical and exam characteristics of the ACRIN 6698 multi-center, external dataset.*

Multi-center external test set	N (%)
**Patients**	221
**Age, years** (mean±SD)	48.1±10.8
**Race**	
White	153 (69.2 %)
Black	18 (8.1 %)
Asian	16 (7.2 %)
Native Hawaiian or other Pacific Islander	0 (0 %)
American Indian or Alaska Native	1 (0.5 %)
Multiple Races	1 (0.5 %)
Unknown	32 (14.5 %)
**T-Stage**	
1	7 (3.2 %)
2	149 (67.4 %)
3	57 (25.8 %)
Unknown	8 (3.6 %)
**HR/HER2 subtype**	
HR+/HER2-	44 (19.9 %)
HR+/HER2+	51 (23.1 %)
HR-/HER2-	89 (40.3 %)
HR-/HER2+	32 (14.5 %)
Unknown	5 (2.3 %)
**Lesion Type**	
Mass(es)	198 (89.6 %)
NME(s)	23 (10.4 %)
**Scanner Manufacturer**	
GE	164 (74.2 %)
Siemens	29 (13.1 %)
Philips	28 (12.7 %)
**Field Strength**	
1.5T	152 (68.8 %)
3T	69 (31.2 %)

Abbreviations: NME: nonmass enhancement, SD: standard deviation.

Table S8: *Detection results for FCDD on the external, multi-center validation test set compared to other models. Cells in bold correspond to HSC or FCDD models that are statistically significant (P<.05) compared to the BCE baseline. All results are reported on the external test set, training on the model development dataset with multiple random seeds.(mean ± SD).*

				Maximizing Youden's Index			Maximizing Sensitivity	
**Task**	**Model**	**AUC**	**AUPR**	**PPV**	**Specificity**	**Sensitivity**	**Specificity @95% Sensitivity**	**Specificity @97% Sensitivity**
**Task 1 Balanced Detection**	BCE	0.79±0.01	0.83±0.01	76%±4 [167/221]	77%±7 [169/221]	71%±7 [156/221]	20%±3 [43/221]	10%±5 [22/221]
	HSC	0.81 ±0.01	0.81±0.01	**78%±2 [172/221]**	**80%±3 [176/221]**	71±5 [157/221]	21%±6 [45/221]	9%±4 [20/221]
	FCDD	**0.86±0.01**	**0.88±0.02**	**82%±0.01 [181/221]**	**83%±1 [183/221]**	**79%±5 [173/221]**	**27%±7 [60/221]**	**12%±3 [26/221]**

Abbreviations: BCE: binary cross entropy, HSC: hypersphere classification, FCDD: fully convolutional data description, AUC: area under the receiver operating curve, AUPR: area under the precision recall curve, PPV: positive predictive value, SD: standard deviation.

Supplemental materials undergo peer review but are not copyedited, and may include typographical errors.

Appendix S1. MRI Acquisition and Postprocessing

Model development dataset

Over the study period (2005 – 2015), two MR imaging protocols, both consistent with American College of Radiology (ACR) breast MR imaging accreditation program guidelines (1) were used as clinical practice and technology evolved. Patients underwent imaging in the prone position, and imaging sequences included T2-weighted and unenhanced and at least two contrast-enhanced T1-weighted fat-suppressed three-dimensional fast spoiled gradient-recalled acquisitions. From July 2005 through January 2010, examinations were performed with a 1.5-T Signa scanner (GE Healthcare, Waukesha, Wis) and dedicated eight-channel breast coil (Sentinelle; Invivo, Gainesville, Fla). DCE images were obtained in the axial plane with the following parameters: repetition time/echo time, 5.5/2.7 msec; flip angle, 10°; field of view, 32–38 cm; section thickness, 1.6 mm; and matrix size, 420 x 420. Initial contrast-enhanced acquisitions were centered at 90 seconds after contrast material administration. From January 2010 to November 2015, examinations were performed with a 3.0-T Achieva TX scanner (Philips Healthcare, Best, the Netherlands) and 16-channel dedicated breast coil (Mammotrak, Philips Healthcare). DCE images were obtained in the axial plane with the following parameters: repetition time/echo time, 5.9/3.0 msec; flip angle, 10°; field of view, 22–33 cm; section thickness, 1.3 mm; and matrix size, 440 x 660. Initial contrast-enhanced acquisitions were centered at 110 seconds after contrast material administration. For all examinations, gadolinium-based contrast material (before November 2010: gadodiamide, Omniscan, GE Healthcare; from November 2010: gadoteridol, ProHance, Bracco Diagnostics, Princeton, NJ) was power injected (0.1 mmol per kilogram of body weight at a rate of 2 mL/sec) and followed by a 20-mL saline flush. Images were processed using a commercially available computer-aided evaluation system (CADstream; Merge Healthcare, Chicago, Ill) to perform motion correction and generate reformats, subtractions, and maximum intensity projections (MIPs; utilized for this study).

Internal test dataset

The internal test dataset comprised patients prospectively enrolled in a breast MRI study (NCT03607552; 2018-2022). MRI scans were performed using the institution's clinical protocol and meeting ACR breast MR imaging accreditation program criteria (1). Patients were imaged in the prone position, and examinations were performed on a 3.0-T Achieva TX imager (Philips Healthcare, Best, the Netherlands) and 16-channel dedicated breast coil (Mammotrak, Philips Healthcare). DCE MRI was obtained in the axial plane with the following parameters: repetition time/echo time, 6.0/3.1 msec; flip angle, 10°; field of view, 24 x 36 cm; section thickness, 1.5 mm; and matrix size, 440 x 660. Initial contrast-enhanced acquisitions were centered at 120 seconds after contrast material administration. Gadolinium-based contrast material (gadoteridol, Prohance, Bracco) was power injected (0.1 mmol per kilogram of body weight at a rate of 2 mL/sec) and followed by a 20-mL saline flush. As described above, subtraction MIPs were generated using the clinical computer-aided evaluation system (CADstream).

External test dataset

The multicenter ACRIN 6698 trial dataset, posted publicly on TCIA [REF] May 2022, was used for external testing and incorporated breast MRI examinations from ten institutions and a variety of MRI scanner platforms. The MRI protocol for the trial has been described previously (2). Briefly, DCE MRI was performed by using a three-dimensional fat-suppressed T1-weighted gradient echo sequence with the following parameters: repetition time, 4–10 msec; echo time, minimum; flip angle, 10–20°; in-plane resolution, ≤1.4 mm; section thickness, ≤2.5 mm; and imaging time, 80–100 seconds. Multiple sequential phases were acquired: one before and five or more (for at least 8 minutes) after intravenous gadolinium-based contrast material injection (0.1 mmol per kilogram of body weight at 2 mL/sec, followed by a 20-mL saline flush). The ACRIN 6698 study protocol did not specify the gadolinium agent to be used but

required that the same agent be used for all MRI examinations for an individual patient. Per the I-SPY 2 trial protocol, tumor volume masks were created by study investigators using a semiautomated software tool (Aegis; Hologic, Danbury, Conn) (2), which were provided on TCIA for each MRI exam.

For this study, motion correction of the DCE MRI sequence was performed using custom software (Matlab, Mathworks Inc) and subtraction MIPs were then generated using the pre- and postcontrast DCE phase acquired closest to 150 seconds after injection (as specified by the trial for tumor volume mask, similar to the clinical MIPs in the internal datasets. A projection of the three-dimensional tumor volume mask was created and used as ground truth tumor regions (ROIs) on corresponding subtraction MIPs for validation of explainability heat maps in this study.

Appendix S2. HSC, FCDD, and FCDD-Symmetric models

FCDD is a variation of the Hypersphere Classification (HSC)(3) (Eq. S1 and S2) which transforms data such that normal samples are concentrated to a predetermined center in the feature space and anomalous data lies elsewhere. For explainability, FCDD maps each breast MIP into an anomaly heatmap; in feature space, the pixels in the heatmap that are far from the data center learned by the model correspond to pixels with high anomaly scores, and the mean score across pixels is used to classify each breast. To achieve this, the FCDD model uses a fully convolutional architecture (FCN)(4,5) and a deep anomaly detection objective adapted from the HSC loss to provide pixel-wise anomaly scores (Section S.I, Eq. S3).

The FCDD model follows the one-class classification paradigm and is an explainable variant of HSC(6) and DSVDD (Deep Support Vector Data Description)(6), the standard approach to one-class classification. In DSVDD, a neural network learns to transform data such that nominal samples are concentrated to a predefined hypersphere center in feature space and anomalous data lies elsewhere. DSVDD trains its network using unlabeled, mostly nominal data. The corresponding loss is:

$$L = \frac{1}{n} \sum_{i=1}^{n} ||f(x_i) \; - \; c \,||^2, \text{ Eq. S1}$$

where $x_1, \ldots, x_n \in R^{h \times w}$ denotes a collection of samples, $f: R^{h \times w} \rightarrow R^d$ is a neural network, and $c \in R^d$ is the hypersphere center. During inference, the loss is small for data like the training data, which are mostly nominal. Thus, the loss is small for nominal data. For atypical, anomalous samples, the loss will be larger, thus making the loss itself an anomaly score.

<u>HSC Model</u>
HSC (Hypersphere Classification) improves upon DSVDD by incorporating known anomalies in the training procedure. It essentially reverses the loss for these anomalies, actively pushing them away from the hypersphere center. In contrast to simple binary classification, HSC still follows the one-class paradigm, thereby taking care that the model is not biased towards the known anomalies, which are underrepresentative of the true anomaly distribution. The HSC loss is:

$$L = \frac{1}{n} \sum_{i=1}^{n} (1 - y_i)\, h(f(x_i) - c) \; - \; y_i \log(1 - \exp(-h(f(x_i) - c))), \text{ Eq. S2}$$

Supplemental materials undergo peer review but are not copyedited, and may include typographical errors.

where $y_1, \ldots, y_n$ are labels with $y_i = 1$ denoting an anomaly and $y_i = 0$ denoting a nominal sample, and $h(a) = \sqrt{||a||_2^2 + 1} - 1$ is the pseudo-Huber loss. HSC is not intrinsically explainable. The anomaly score is merely a scalar, and it is unknown to which regions of the input the values in the feature vector $f(x_i)$ correspond to.

FCDD Model

FCDD makes the HSC model explainable by transforming the input image into a full anomaly heatmap instead of a feature vector. Each pixel in this heatmap is an anomaly score on its own, rating the anomalousness of the respective region in the input. To achieve this, FCDD employs a fully convolutional network $f: R^{h \times w} \to R^{u \times v}$ (FCN)(4) where each pixel in the output has a limited receptive field in the input. Again, utilizing the pseudo-Huber loss and redefining $c \in R^{u \times v}$, the anomaly heatmap is defined entry-wise as $A(x) = \sqrt{(f(x) - c)^2 + 1} - 1$. The final FCDD objective is:

$$L = \frac{1}{n} \sum_{i=1}^{n} (1 - y_i) \frac{1}{u \cdot v} \left|\left|A(x_i)\right|\right|_1 - y_i \log\left(1 - \exp\left(- \frac{1}{u \cdot v} \left|\left|A(x_i)\right|\right|_1\right)\right), \text{ Eq. S3}$$

Here $\left|\left|A(x)\right|\right|_1$ is the sum of all entries in $A(x)$, which are all positive. The objective maximizes $\left|\left|A(x)\right|\right|_1$ for anomalous samples and minimizes it for nominal samples. In consequence, $\left|\left|A(x)\right|\right|_1$ becomes an anomaly score. Entries of $A(x)$ that contribute to $\left|\left|A(x)\right|\right|_1$ correspond to regions of the input image that add to the anomaly score. Hence, pixels in this heatmap that have large values correspond to anomalous regions in the input. Since $A(x)$ is typically of smaller resolution than the original input ($u < h, v < w$), it is upsampled to the original input image resolution using a strided transposed convolution with a fixed Gaussian kernel.

Results for these models are summarized in Tables S1 and S2 for various training settings.

Statistical tests comparing FCDD to BCE are presented in Table S3.

FCDD-Symmetric Model

We also explore an adaptation of FCDD that takes advantage of the breasts being symmetric to each other. For each scan of a breast x_i there exists a nominal counterpart z_i. We can use this nominal counterpart to learn a hypersphere center via a neural network $g: R^{h \times w} \to R^d$. Redefining FCDD's FCN as $f: R^{h \times w} \to R^{u \times v \times d}$, the anomaly heatmap becomes:

$$A(x) = \sqrt{\left(f(x_i) - \mathbf{1}^{u \times v} \otimes g(z_i)\right)^2 + 1} - 1., \text{ Eq. S4}$$

Here the term $g(z_i)$ produces a vector from one of the breasts and is meant to represent the general properties of the breast under consideration so that anomalousness is with respect to the other breast. The tensor product $\mathbf{1}^{u \times v} \otimes g(z_i)$ simply stacks $u \times v$ copies of $g(z_i)$ so now the anomaly score per pixel is with respect to the distance from $g(z_i)$ rather than zero. Using the FCDD objective from above with this new anomaly heatmap yields a new objective, which we call "FCDD-Symmetric". At training time, we define the nominal sample as the contralateral breast if the contralateral breast is benign, if the contralateral breast is malignant or is not available, we replace it with a benign, randomly sampled breast.

Supplemental materials undergo peer review but are not copyedited, and may include typographical errors.

At inference time, we pass both the test breast and the contralateral image as reference, regardless of its diagnosis to avoid data leakage.

Appendix S3. Network architectures, hyperparameters and visualization

For comparison to the proposed FCDD model, we used two models with VGG-11 backbones with comparable number of parameters and depth. The first model used binary cross entropy (BCE), the traditional loss for binary classification. The second model was the Hypersphere Classification (HSC)(3) model, a non-explainable anomaly detection model. For comparison in explainability, we computed the Gradient-weighted Class Activation Mapping (Grad-CAM)(7) saliency map for both BCE and HSC models, a common interpretability technique in convolutional neural networks, which has been demonstrated to be more robust than other saliency methods in medical imaging.(8)

Our architecture and training parameters are based in the Liznerski et al., 2021 with modifications detailed below. The results of Liznerski et al., 2021 were independently reproduced and confirmed by *Bertoldo, J. P., & Decencière, E. (2022). [Reproducibility Report] Explainable Deep One-Class Classification. arXiv preprint arXiv:2206.02598.*

BCE and HSC architecture (VGG 11 with batch normalization):
BCE and HSC share the same network architecture, a VGG 11 network modified with batch normalization. The network is described below:

```
VGG_CROP(
 (core): Sequential(
  (0): Conv2d(3, 64, kernel_size=(3, 3), stride=(1, 1), padding=(1, 1))
  (1): BatchNorm2d(64, eps=1e-05, momentum=0.1, affine=True, track_running_stats=True)
  (2): ReLU(inplace=True)
  (3): MaxPool2d(kernel_size=2, stride=2, padding=0, dilation=1, ceil_mode=False)
  (4): Conv2d(64, 128, kernel_size=(3, 3), stride=(1, 1), padding=(1, 1))
  (5): BatchNorm2d(128, eps=1e-05, momentum=0.1, affine=True, track_running_stats=True)
  (6): ReLU(inplace=True)
  (7): MaxPool2d(kernel_size=2, stride=2, padding=0, dilation=1, ceil_mode=False)
  (8): Conv2d(128, 256, kernel_size=(3, 3), stride=(1, 1), padding=(1, 1))
  (9): BatchNorm2d(256, eps=1e-05, momentum=0.1, affine=True, track_running_stats=True)
  (10): ReLU(inplace=True)
  (11): Conv2d(256, 256, kernel_size=(3, 3), stride=(1, 1), padding=(1, 1))
  (12): BatchNorm2d(256, eps=1e-05, momentum=0.1, affine=True, track_running_stats=True)
  (13): ReLU(inplace=True)
  (14): MaxPool2d(kernel_size=2, stride=2, padding=0, dilation=1, ceil_mode=False)
  (15): Conv2d(256, 512, kernel_size=(3, 3), stride=(1, 1), padding=(1, 1))
  (16): BatchNorm2d(512, eps=1e-05, momentum=0.1, affine=True, track_running_stats=True)
  (17): ReLU(inplace=True)
  (18): Conv2d(512, 512, kernel_size=(3, 3), stride=(1, 1), padding=(1, 1))
  (19): BatchNorm2d(512, eps=1e-05, momentum=0.1, affine=True, track_running_stats=True)
  (20): ReLU(inplace=True)
  (21): MaxPool2d(kernel_size=2, stride=2, padding=0, dilation=1, ceil_mode=False)
  (22): Conv2d(512, 512, kernel_size=(3, 3), stride=(1, 1), padding=(1, 1))
```

Supplemental materials undergo peer review but are not copyedited, and may include typographical errors.

```
    (23): BatchNorm2d(512, eps=1e-05, momentum=0.1, affine=True, track_running_stats=True)
    (24): ReLU(inplace=True)
    (25): Conv2d(512, 512, kernel_size=(3, 3), stride=(1, 1), padding=(1, 1))
    (26): BatchNorm2d(512, eps=1e-05, momentum=0.1, affine=True, track_running_stats=True)
    (27): ReLU(inplace=True)
    (28): MaxPool2d(kernel_size=2, stride=2, padding=0, dilation=1, ceil_mode=False)
  )
    (1): MaxPool2d(kernel_size=2, stride=2, padding=0, dilation=1, ceil_mode=False)
    (2): AdaptiveAvgPool2d(output_size=7)
    (3): Flatten(start_dim=1, end_dim=-1)
  )
  (lin): Linear(in_features=25088, out_features=1, bias=True)
)

==========================================================
Total params: 5,538,049
Trainable params: 5,538,049
Non-trainable params: 0
```

FCDD Architecture

The FCDD architecture is a fully convolutional architecture. It does not contain any fully connected layers nor global pooling. The output feature map is up sampled with the strided transposed algorithm with Gaussian kernel (std=10) described in Liznerski et al., 2021.

```
FCDD_CNN224_VGG_NOPT(
  (features): Sequential(
    (0): RecConv2d(3, 64, kernel_size=(3, 3), stride=(1, 1), padding=(1, 1))
    (1): BatchNorm2d(64, eps=1e-05, momentum=0.1, affine=True, track_running_stats=True)
    (2): ReLU(inplace=True)
    (3): RecMaxPool2d(kernel_size=2, stride=2, padding=0, dilation=1, ceil_mode=False)
    (4): RecConv2d(64, 128, kernel_size=(3, 3), stride=(1, 1), padding=(1, 1))
    (5): BatchNorm2d(128, eps=1e-05, momentum=0.1, affine=True, track_running_stats=True)
    (6): ReLU(inplace=True)
    (7): RecMaxPool2d(kernel_size=2, stride=2, padding=0, dilation=1, ceil_mode=False)
    (8): RecConv2d(128, 256, kernel_size=(3, 3), stride=(1, 1), padding=(1, 1))
    (9): BatchNorm2d(256, eps=1e-05, momentum=0.1, affine=True, track_running_stats=True)
    (10): ReLU(inplace=True)
    (11): RecConv2d(256, 256, kernel_size=(3, 3), stride=(1, 1), padding=(1, 1))
    (12): BatchNorm2d(256, eps=1e-05, momentum=0.1, affine=True, track_running_stats=True)
    (13): ReLU(inplace=True)
    (14): RecMaxPool2d(kernel_size=2, stride=2, padding=0, dilation=1, ceil_mode=False)
    (15): RecConv2d(256, 512, kernel_size=(3, 3), stride=(1, 1), padding=(1, 1))
    (16): BatchNorm2d(512, eps=1e-05, momentum=0.1, affine=True, track_running_stats=True)
    (17): ReLU(inplace=True)
    (18): RecConv2d(512, 512, kernel_size=(3, 3), stride=(1, 1), padding=(1, 1))
    (19): BatchNorm2d(512, eps=1e-05, momentum=0.1, affine=True, track_running_stats=True)
    (20): ReLU(inplace=True)
  )
```

Supplemental materials undergo peer review but are not copyedited, and may include typographical errors.

```
 (conv_final): RecConv2d(512, 1, kernel_size=(1, 1), stride=(1, 1))
)

================================================================
Total params: 4,504,833
Trainable params: 4,504,833
Non-trainable params: 0
Receptive filed (pixels): 62 x 62
```

Preprocessing and hyperparameters

After MIP images were created, MATLAB (Mathworks, Natick, MA) was used to preprocess MIPs for model training and evaluation, as previously described (9). Each image was cropped to split the MIP into two single-breast images, and then the top 0.5% of pixel intensities of each image were clipped, assuming these extreme values represented noise due to artifacts. Lastly, each image was resized to 224 x 224 pixels.

At training time, the images were preprocessed in the following sequence using *pytorch* and *torchvision*: (1) resize from 224x224 pixels to 256x256 pixels, (2) random color jitter (0.01 brightness, contrast, saturation , hue), (3) random horizontal flip with 50% chance to reduce effect of breast orientation, (3) center crop to 224x224, (4) additive Gaussian noise with σ=0.001 and (5) data normalization. At inference time, we use only center crop and standardization.

Hyperparameters were chosen based on the ImageNet parameters used in Liznerski et al., 2021. We trained all models for 200 epochs, batch size 32, SGD with weight decay 1e-06, Nesterov momentum μ=0.9, learning rate of 0.001 with decay 0.985. We performed grid search on an internal validation split in the first fold to define the final number of epochs, learning rate and momentum parameters. No other hyperparameter was tuned as the original model shows good generalization across different datasets.

Heatmap visualization

For comparable anomaly heatmap visualization, the heatmap pixels are scaled to values in [0,1]. Instead of min-max scaling, we use anomaly score quantiles to adjust the contrast in the heatmaps according to Liznerski et al., 2021. For all maps in this work, we choose the normalization quantiles as 0.97. The pixels can be normalized across all cases (global normalization) or across each image (local normalization). We found the latter more useful when comparing to the radiologist and specify in the main manuscript if global or local normalization is used in every instance.

Appendix S4. HSC results

HSC is observed to have good detection performance in both tasks, as summarized Table 2.

Figure S1 and Figure S2, also included in Figure 4a and 4b, present examples of gradient-based saliency maps for HSC. The explanation heatmaps of HSC seems to be like those of FCDD and superior to the BCE heatmaps, but they tend to select multiple small anomalous regions whereas FCDD tends to select a single region in most images. When compared to the radiologist annotations, Figure S3, the HSC heatmaps have good performance compared to BCE but some small lesions are missed (Stage 1, last image on the right) and some large lesions are underestimated (Stage 3, last image on the right). Overall, HSC demonstrates good explainability.

Supplemental materials undergo peer review but are not copyedited, and may include typographical errors.

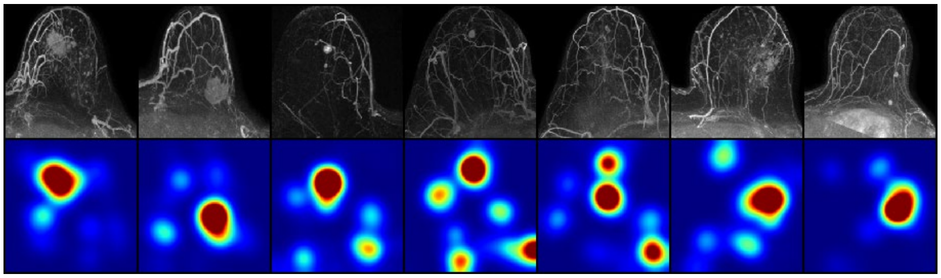

Figure S1: HSC Task 1 saliency maps computed using Grad-CAM. Cases are the same as Figure 4a.

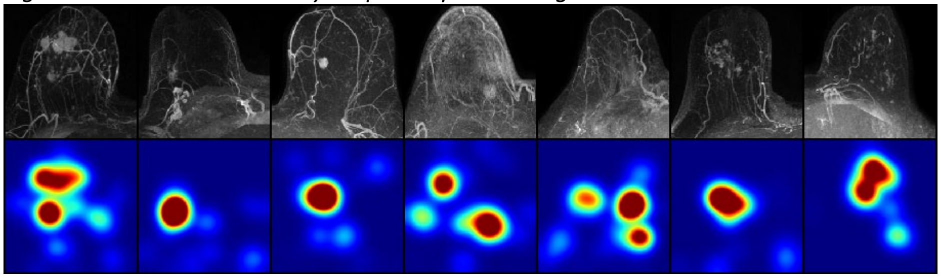

Figure S2: HSC Task 2 saliency maps computed using Grad-CAM. Cases are the same as Figure 4b.

Supplemental materials undergo peer review but are not copyedited, and may include typographical errors.

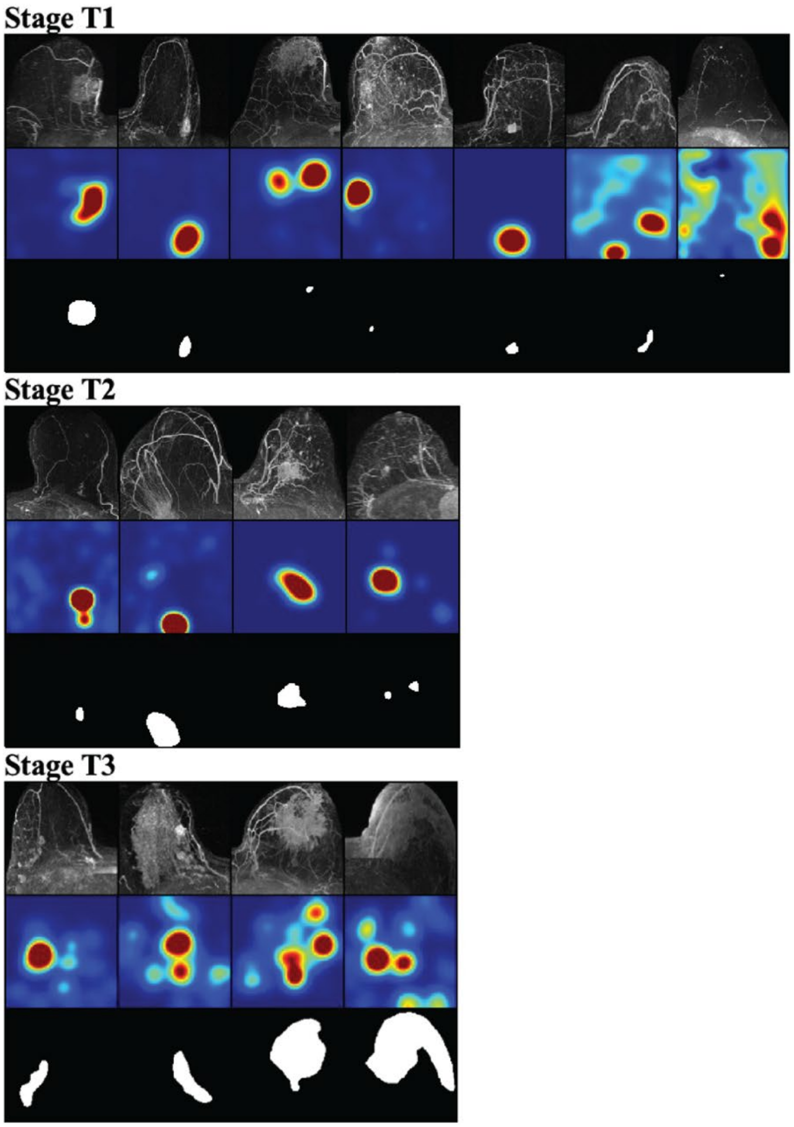

Figure S3: HSC saliency maps computed using Grad-CAM and radiologist annotations for groups of cases of stage T categories T1, T2 and T3, respectively. Cases are the same as Figure 5e. The saliency maps seem to be more accurate than those for BCE, but less accurate than FCDD in some cases.

Appendix S5. FCDD-Symmetric results

Table S4 compares the detection performance of FCDD-Symmetric compared to other models. We observe FCDD outperform BCE, but it tends to have higher variance than FCDD and HSC in most metrics. Thus, FCDD-Symmetric is not necessarily better compared to FCDD.

Figure S4 presents the pixel-wise AUC explanation scores for the FCDD-Symmetric model. We observe that FCDD-Symmetric has a higher median that the rest of model explanations, but it has higher variance.

Supplemental materials undergo peer review but are not copyedited, and may include typographical errors.

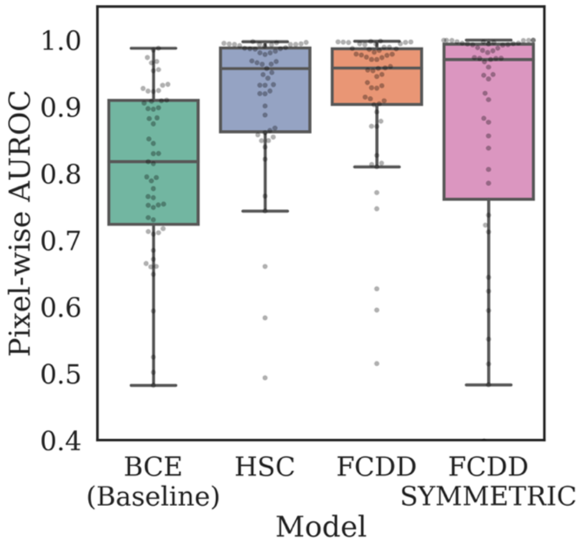

Figure S4: Pixelwise AUC for each case in the explanation compared to radiologist annotations.

The results of multiple Wilcoxon tests for each model in the explanation cohort are presented below. FCDD-Symmetric explanations are not statistically significant compared to BCE explanations, albeit is higher median performance.

Wilcoxon BCE vs FCDD
statistic = 143.0, p value = 1.49e-07
Wilcoxon BCE vs HSC
statistic = 179.0, p value = 7.36e-07
Wilcoxon BCE vs FCDD-Symmetric
statistic = 538.0, p value = 0.0519

Appendix S6. Validation on external, multi-center test set

As exploratory analysis, we evaluated the model on a sample of the ACRIN 6698 trial dataset. Table S6 summarizes the clinical characteristics of this cohort. We observe that this is a balanced dataset comprised of 221 malignant breasts and 221 contralateral benign breasts, per our definitions in the Materials and Methods. We computed MIPs from the DCE-MRI and applied the same pre-processing as done in the internal dataset. Since the lesions were segmented in each slice of the MRI, we projected this lesion segmentation into the MIP as our ground-truth lesion segmentations. Once the dataset was processed, we evaluated the FCDD, HSC and BCE models in zero-shot fashion (no retraining with ACRIN 6698 data) on this external test set. All models were trained in all of our internal data and 5 random seeds were used per model to report standard deviation in predictions.

As presented in Table S7, FCDD had a zero-shot detection performance that was substantially higher than HSC and BCE (P<.001 in both cases). In this balanced detection setting, FCDD presented (AUC=0.863±0.01, AUPR=0.88±0.02) that was substantially higher (P<.001) than HSC (AUC=0.806±0.01, AUPR=0.809±0.01)

Supplemental materials undergo peer review but are not copyedited, and may include typographical errors.

and the BCE model (AUC=0.792±0.01, AUPR=0.825 ± 0.01). Other classification metrics are summarized in Table S7 and the Results section, leading to the same conclusion.

Also, as presented in Figure S5, the quality of explanation maps was substantially higher for FCDD. Our model had a pixel-wise AUC of 0.847±0.13 in the external test set compared to BCE ($P<.001$, pixel-wise AUC=0.551±0.12) and HSC ($P<.001$, pixel-wise AUC=0.756±0.15).

These results confirm the generalization and high performance of the FCDD model for both detection and explanation tasks.

Supplemental materials undergo peer review but are not copyedited, and may include typographical errors.

a) Pixel-wise AUROC on ACRIN 6689 external test set (zero-shot)

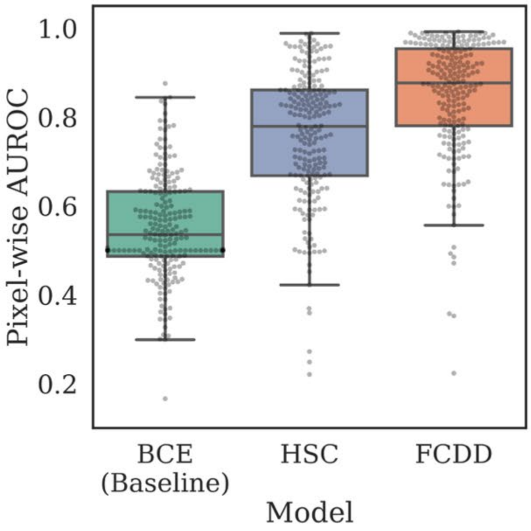

b) Random sample of ACRIN 6689 MIPs, comparing model explanations and radiologist annotations

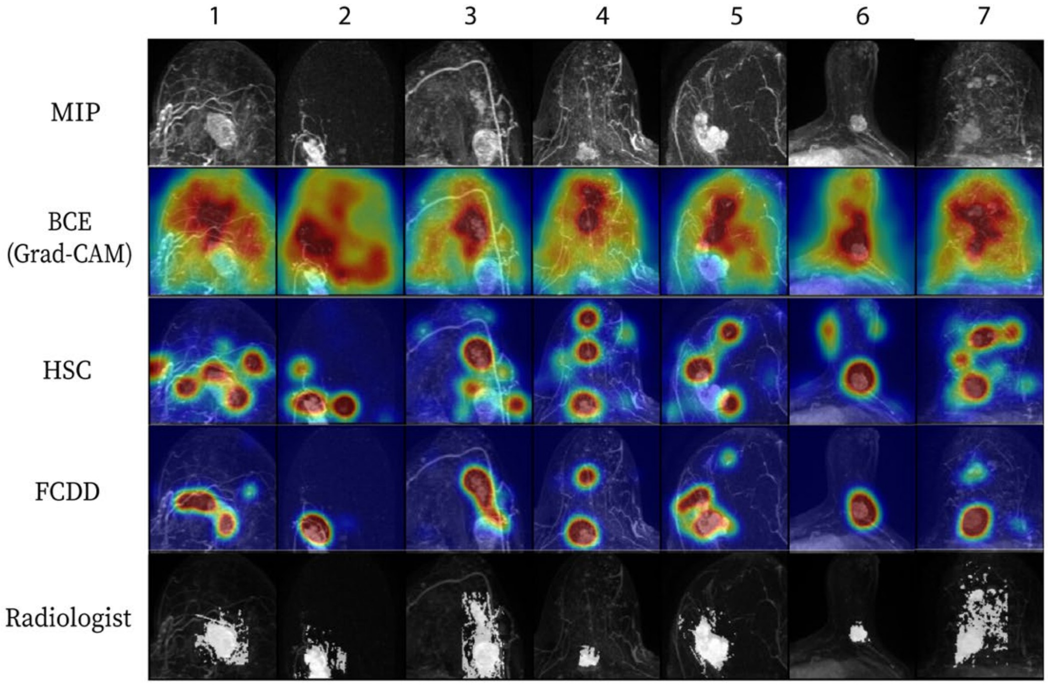

Figure S5: ***Validation of model explanations in the external multi-center dataset (ACRIN 6689).*** *a) Pixelwise AUC for various models using the radiologist annotation as ground truth. Each point in the boxplot corresponds to the pixelwise AUC between a saliency heatmap and the radiologist annotation in a specific breast image. Model prediction is done in zero-shot fashion in the external dataset (no re-training). b) Random sample of cases comparing the radiologist annotation to BCE (Grad-CAM), HSC and FCDD explanation maps. All maps are locally normalized.*

Supplemental materials undergo peer review but are not copyedited, and may include typographical errors.

References

1. MRI Exam-Specific Parameters: Breast (Revised 3-19-2025). Accreditation Support. https://accreditationsupport.acr.org/support/solutions/articles/11000114407-mri-exam-specific-parameters-breast-revised-3-19-2025-. Accessed May 1, 2025.

2. ACRIN 6698 Trial Team and I-SPY 2 Trial Investigators, Partridge SC, Zhang Z, et al. Diffusion-weighted MRI findings predict pathologic response in neoadjuvant treatment of breast cancer: The ACRIN 6698 multicenter trial. RADIOLOGY. 2018;289(3):618–627. doi: 10.1148/radiol.2018180273.

3. Ruff L, Vandermeulen RA, Franks BJ, Müller K-R, Kloft M. Rethinking Assumptions in Deep Anomaly Detection. arXiv; 2023. http://arxiv.org/abs/2006.00339. Accessed December 28, 2023.

4. Long J, Shelhamer E, Darrell T. Fully Convolutional Networks for Semantic Segmentation. .

5. Sabokrou M, Fayyaz M, Fathy M, Moayedd Z, klette R. Deep-Anomaly: Fully Convolutional Neural Network for Fast Anomaly Detection in Crowded Scenes. arXiv; 2017. http://arxiv.org/abs/1609.00866. Accessed December 28, 2023.

6. Ruff L, Vandermeulen R, Goernitz N, et al. Deep One-Class Classification. Proceedings of the 35th International Conference on Machine Learning. PMLR; 2018. p. 4393–4402. https://proceedings.mlr.press/v80/ruff18a.html. Accessed December 28, 2023.

7. Selvaraju RR, Cogswell M, Das A, Vedantam R, Parikh D, Batra D. Grad-CAM: Visual Explanations From Deep Networks via Gradient-Based Localization. .

8. Saporta A, Gui X, Agrawal A, et al. Benchmarking saliency methods for chest X-ray interpretation. Nat Mach Intell. Nature Publishing Group; 2022;4(10):867–878. doi: 10.1038/s42256-022-00536-x.

9. Holste G, Partridge SC, Rahbar H, Biswas D, Lee CI, Alessio AM. End-to-End Learning of Fused Image and Non-Image Features for Improved Breast Cancer Classification from MRI. 2021 IEEE/CVF International Conference on Computer Vision Workshops (ICCVW). Montreal, BC, Canada: IEEE; 2021. p. 3287–3296. doi: 10.1109/ICCVW54120.2021.00368.
